# SPIONs mediated magnetic actuation promotes nerve regeneration by inducing and maintaining repair-supportive phenotypes in Schwann cells

**DOI:** 10.1186/s12951-022-01337-5

**Published:** 2022-03-27

**Authors:** Ting Liu, Yang Wang, Laijin Lu, Yi Liu

**Affiliations:** 1 Department of Geriatrics, The First Hospital of Jilin University, Changchun, 130021 People’s Republic of China; 2 Department of Hand Surgery, The First Hospital of Jilin University, Changchun, 130021 People’s Republic of China; 3 State Key Laboratory of Supramolecular Structure and Materials, College of Chemistry, Jilin University, Changchun, 130012 People’s Republic of China

**Keywords:** Repair Schwann cell, Repair phenotypes, Nerve regeneration, Superparamagnetic iron oxide nanoparticles (SPIONs), Magnetic actuation, Mechanotransduction

## Abstract

**Background:**

Schwann cells, the glial cells in the peripheral nervous system, are highly plastic. In response to nerve injury, Schwann cells are reprogrammed to a series of specialized repair-promoting phenotypes, known as repair Schwann cells, which play a pivotal role in nerve regeneration. However, repair Schwann cells represent a transient and unstable cell state, and these cells progressively lose their repair phenotypes and repair‐supportive capacity; the transience of this state is one of the key reasons for regeneration failure in humans. Therefore, the ability to control the phenotypic stability of repair Schwann cells is of great practical importance as well as biological interest.

**Results:**

We designed and prepared a type of fluorescent–magnetic bifunctional superparamagnetic iron oxide nanoparticles (SPIONs). In the present study, we established rat sciatic nerve injury models, then applied SPIONs to Schwann cells and established an effective SPION-mediated magnetic actuation system targeting the sciatic nerves. Our results demonstrate that magnetic actuation mediated by SPIONs can induce and maintain repair-supportive phenotypes of Schwann cells, thereby promoting regeneration and functional recovery of the sciatic nerve after crush injury.

**Conclusions:**

Our research indicate that Schwann cells can sense these external, magnetically driven mechanical forces and transduce them to intracellular biochemical signals that promote nerve regeneration by inducing and maintaining the repair phenotypes of Schwann cells. We hope that this study will provide a new therapeutic strategy to promote the regeneration and repair of injured peripheral nerves.

**Graphical Abstract:**

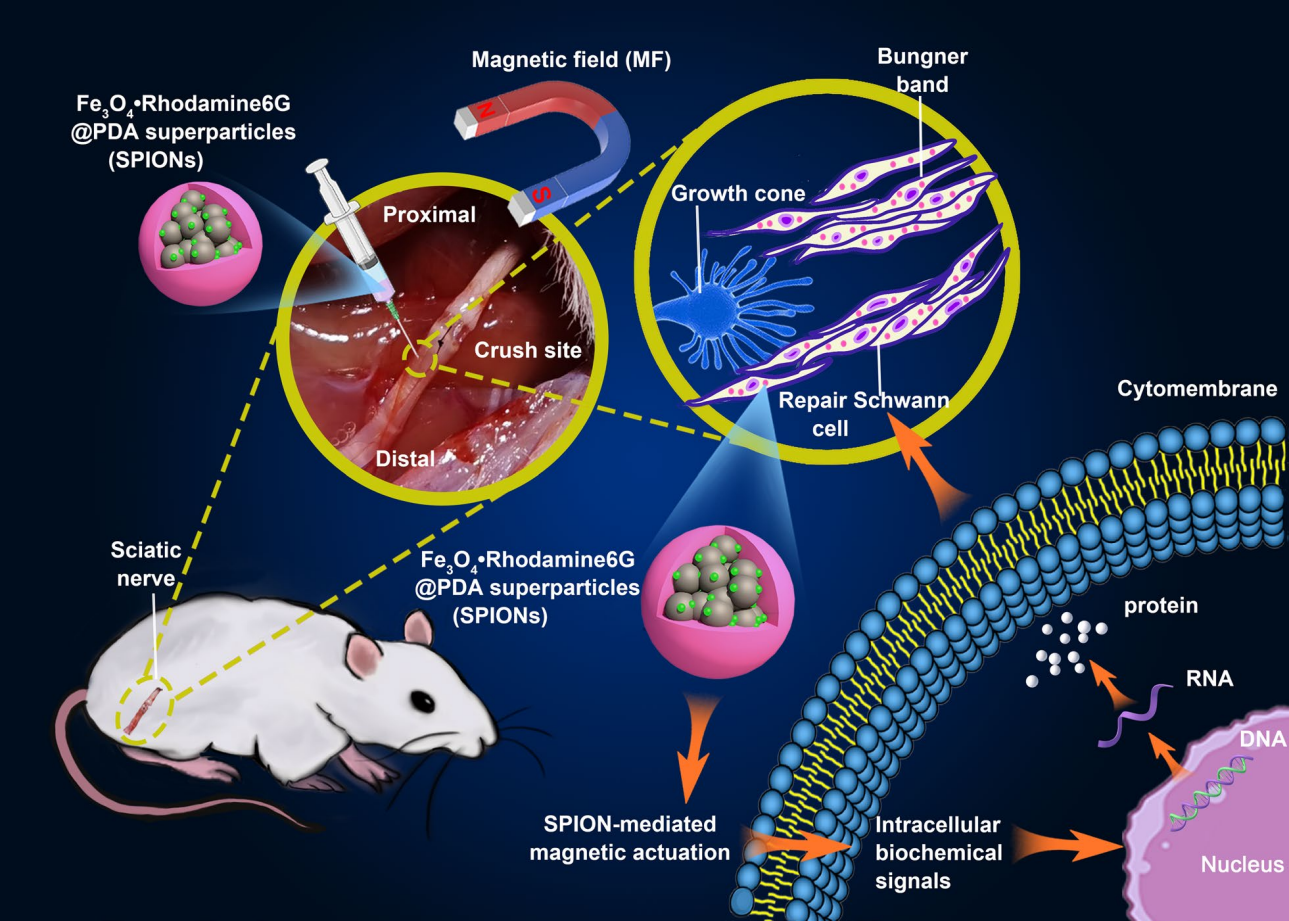

**Supplementary Information:**

The online version contains supplementary material available at 10.1186/s12951-022-01337-5.

## Background

Peripheral nerve injury is one of the most frequently encountered clinical conditions in neurology, resulting in the loss of motor, sensory and autonomic functions in denervated limbs. Despite progress in surgical intervention, the outcomes of peripheral nerve injury remain unsatisfactory [1, 2]. Peripheral nerve injury triggers a series of well-defined events both proximal and distal to the injury site. Axonal disconnection causes changes of molecular and cellular functional states in the soma of the neuron, which lead to activation of regeneration-related genes [3, 4]. Axons distal to the injury degenerate, Schwann cells convert to repair-supportive phenotypes, and macrophages enter the nerve to clear myelin and axonal debris. Following these events, axons must regrow through the distal part of the nerve, reinnervate target organs and finally be remyelinated by Schwann cells. The primary treatment strategy for peripheral nerve injury is to bridge the lesion by promoting axon regeneration. Therefore, understanding the mechanism of axon regeneration and guiding its growth is essential to improve the functional prognosis of patients with nerve injury.

Schwann cells, the glial cells in the peripheral nervous system that form the myelin sheath around neuronal axons, are critical for the propagation of action potentials and protect the integrity of nerve fibers [5, 6]. Schwann cells are highly plastic cells, and upon injury, they are reprogrammed to a series of specialized repair-promoting phenotypes, which occupy the nerve distal to the injury site, support the survival of axotomized neurons, provide biochemical signals and spatial cues promoting axonal regeneration, organize myelin clearance, and form regeneration tracks (bands of Bungner) that guide axons back to their targets [7–11]. Since these cells are specialized for repair after nerve injury and differ from other cells in the Schwann cell lineage (myelin and Remak states), we refer to these Schwann cells with special repair supporting phenotypes as repair Schwann cells [5, 12–15].

Peripheral nerves have regenerative potential due to the flexible differentiation state of Schwann cells and their ability to convert to cells devoted to repair after injury. As one might expect, the regeneration of injured nerves depends on the response of Schwann cells to injury; Schwann cells have been the focus of research in the field of nerve regeneration therapy due to their pivotal role in nerve injury repair. However, repair Schwann cells represent a transient and unstable cell state meeting the particular demands that arise in injured tissue, and over time, these cells progressively fail to support axon regeneration [5, 16]. Repair Schwann cells gradually lose their repair phenotypes and repair-supportive capacity; this decline is one of the key reasons for regeneration failure in humans [9, 17–20]. Maintaining the repair-supporting features of repair Schwann cells is a central issue for the success of nerve regeneration, and the ability to control the phenotypic stability of repair cells is therefore of great practical importance as well as biological interest. Analysis of the cell-extrinsic and cell-intrinsic pathways that maintain the repair phenotypes and the identification of pharmacological tools and cellular therapeutic approaches that promote nerve regeneration by modulating the cellular and molecular biology of repair Schwann cells are clearly important future research directions [21–24].

**Scheme 1.**
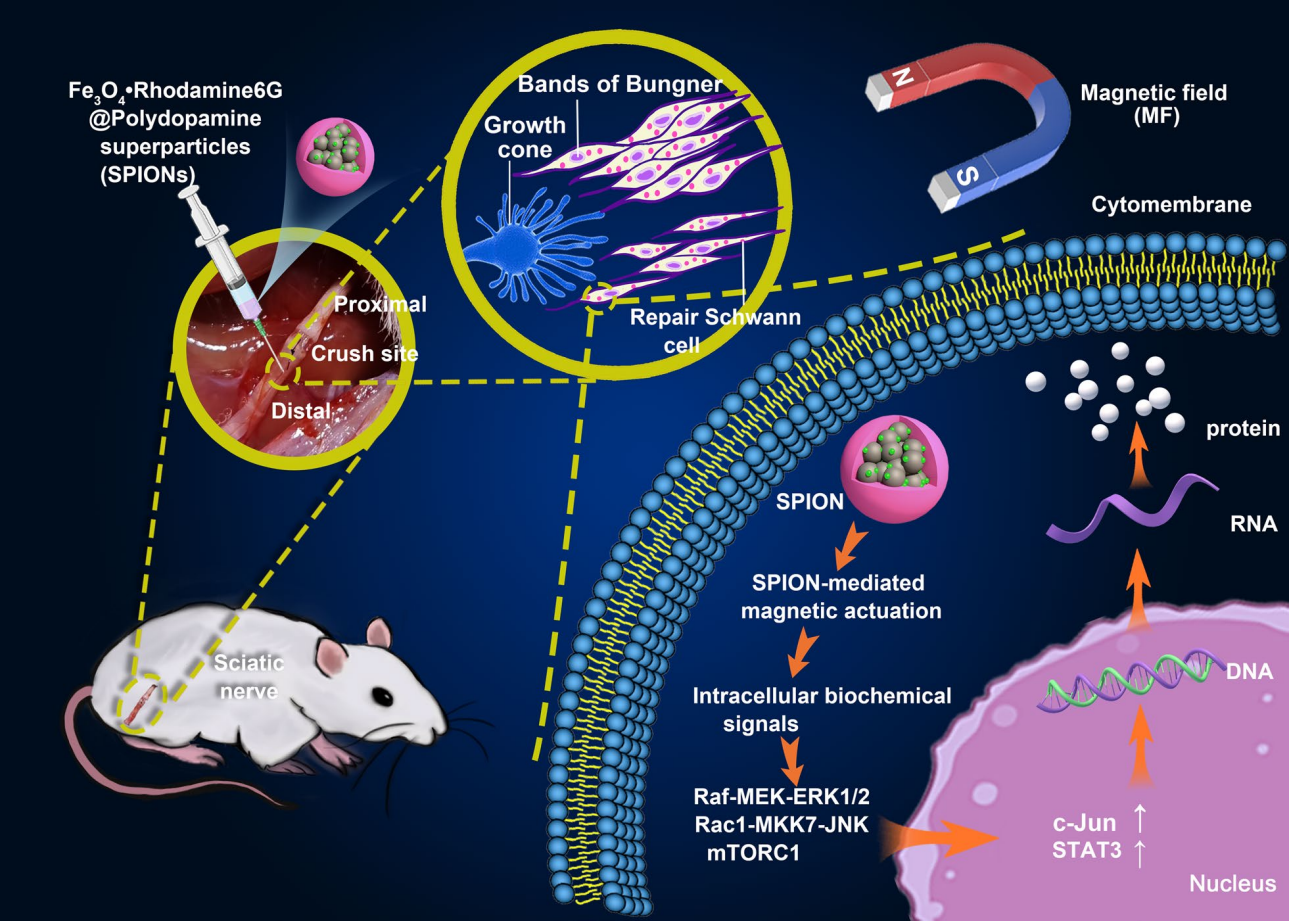
Schematic illustration of SPION-mediated magnetic actuation promoting nerve regeneration by inducing and maintaining repair-supportive phenotypes in Schwann cells. SPIONs were injected under the epineurium at the distal end of the sciatic nerve crush site, then the sciatic nerve was exposed at the gradient magnetic field (MF). Through interacting with magnetic field, SPIONs produce a magnetic stimulation on Schwann cells, initiating the mechanotransduction process, activating intracellular biochemical signals, inducing and maintaining the repair phenotypes of Schwann cells by regulating the expression of regeneration-related genes, and ultimately promoting sciatic nerve regeneration

In recent studies, the influence of mechanical stimuli on the cellular and molecular biology of Schwann cells has been increasingly recognized [25]. Superparamagnetic iron oxide nanoparticles (SPIONs) offer attractive possibilities in biomedicine and can be incorporated into cells, affording a safe and reliable means of influencing the behavior of the cells through magnetic actuation [26]. In our previous research, we designed and prepared a novel type of fluorescent-magnetic bifunctional SPION (specifically, Fe_3_O_4_·Rhodamine 6G@polydopamine superparticle) to drive Schwann cell targeting via magnetic forces [27]. In this study, we applied SPIONs to the sciatic nerve and established a magnetic field (MF) stimulation system for magnetic actuation of Schwann cells. Our results indicate that magnetically driven, SPION-mediated mechanical forces can be sensed by Schwann cells and transduced into intracellular biochemical signals that promote nerve regeneration by inducing and maintaining the repair phenotypes of Schwann cells (Scheme 1).

## Methods

### Materials and cell culture

The RSC96 cells used in this work were obtained from the Shanghai Cell Bank, Chinese Academy of Sciences, and cultured in Dulbecco’s modified Eagle’s medium (DMEM) supplemented with 10% fetal bovine serum (FBS) and 1% antibiotics (100 U/mL penicillin and 100 μg/mL streptomycin). FBS, phosphate buffered solution (PBS), DMEM, penicillin–streptomycin, and trypsin–EDTA were obtained from Life Technologies Corporation (29851 Willow Creek Road, Eugene, OR 97402, USA).

Herein, to study the influence of magnetic actuation on the repair phenotypes of Schwann cells, we cultured RSC96 cells (rat Schwann cells, a type of neuroglial cells in peripheral nervous system) on 35 mm imaging ibidi petri dishes (ibidi, 80156, Martinsried, Germany). For in vitro cell experiments, five experimental groups were designed and established: (1) the normal control group (labeled the ‘Normal’ group and consisting of RSC96 cells cultured under normal conditions, without SPIONs and with a null MF), (2) the magnetic actuation group (labeled the ‘SPIONs + MF’ group and consisting of RSC96 cells treated with both SPIONs and a MF), (3) the positive control group (labeled the ‘c-Jun’ group and consisting of RSC96 cells treated with 0.2 µM anisomycin, which activates the JNK pathway and its downstream c-Jun transcriptional regulation mechanism and then induces the repair phenotypes in RSC96 cells), (4) the SPIONs control group (labeled the ‘SPIONs’ group and consisting of RSC96 cells treated with SPIONs and no MF), and (5) the MF control group (labeled the ‘MF’ group and consisting of cells treated with an MF but no SPIONs).

The c-Jun is a major phosphorylation target of c-Jun N-terminal kinase (JNK). Anisomycin is a potent specific agonist of JNK at a concentration of 0.2 µM [28]. In the positive control group, anisomycin was used to activate JNK, thereby activating the expression of the transcription factor c-Jun, which is critical for inducing the repair function of Schwann cells [12]. The c-Jun-activated Schwann cells were used as the positive control for repair Schwann cells. All cultures were maintained in an incubator at 37 °C in a humidified atmosphere with 5% CO_2_.

### Synthesis and characterization of fluorescent-magnetic bifunctional SPIONs

The fluorescent-magnetic bifunctional SPIONs used in our study (Fe_3_O_4_·Rhodamine 6G@polydopamine superparticles) were prepared following our previously established protocol [27, 29]. In brief, Fe_3_O_4_ nanoparticles (NPs) with average diameter of 5.8 nm were synthesized following the classical thermal decomposition method. Subsequently, Fe_3_O_4_ NPs were mixed with Rhodamine 6G to obtain Fe_3_O_4_·Rhodamine 6G superparticles (SPs) with an average diameter of 50 nm. Finally, polydopamine (PDA) was coated on the surface of Fe_3_O_4_·rhodamine 6G SPs to obtain Fe_3_O_4_· Rhodamine 6G@PDA SPs (SPIONs). After the synthesis of SPIONs, their physical, optical and magnetic properties were characterized respectively. The detailed preparation of SPIONs is presented in the Additional file 1.

### In vivo biodistribution and biocompatibility of SPIONs

To evaluate the in vivo toxicities and biodistribution of SPIONs, rats were intravenously injected with SPIONs at a dose of 1 mg/kg body weight through the caudal vein (200 µL). The normal control group was injected with 200 μL normal saline through the caudal vein. The animals were sacrificed by inhalation of carbon dioxide followed anesthetized at various time points (1, 2, 3, 7 and 14 days) after intravenous injection, and the aorta was approached through an abdominal incision and cannulated just distal to the emergence of the renal arteries. Blood samples were collected from each rat for serum biochemical measurements, including aspartate transaminase (AST), alanine transaminase (ALT), alkaline phosphatase (ALP), total protein (TP), albumin (ALB), and creatinine (Cr). After the blood samples were collected, 500 mL of buffered normal saline was injected, and the right atrium was transected to permit drainage of the blood and injected solution to clean the vascular beds of all major organs of the body. Next, the major organs (heart, liver, spleen, lung, kidney, and brain) were harvested and washed twice with cold PBS. A portion of the organs was collected and fixed in 4% paraformaldehyde (PFA) for histopathological analysis. For conventional histopathological analysis, the major organs of rats were collected, fixed, dehydrated, embedded in paraffin, sectioned, stained with hematoxylin and eosin (HE), and examined using a digital optical microscope (BX51, Olympus Corporation, Tokyo, Japan). The remaining organs were accurately weighed (W_tissue_) and used for in vivo biodistribution evaluation. The amount of iron inside the different organs (W_Fe_) was measured by inductively coupled plasma-atomic emission spectrometry (ICP-AES) with a PerkinElmer Optima 3300DV. The iron content per gram of the organ (relative weight, W_Fe_/W_tissue_) can be obtained by dividing the mass of iron in the organ (W_Fe_) by the mass of the organ (W_tissue_). By subtracting the W_Fe_/W_tissue_ in the normal control group from the W_Fe_/W_tissue_ at different time points (1, 2, 3, 7 and 14 days) after injection, the quantity of exogenous iron from intravenous injection (SPIONs) in major organs can be obtained.

In order to further study the neurotoxicity and neuronal affinity of SPIONs, 300 µg/mL SPIONs (20 µL) was injected locally under the epineurium of the sciatic nerve. At various points (1, 3, 7, and 14 days) following local injection of the sciatic nerve, nerve tissue was collected for histopathological analysis to identify possible neurotoxicity of SPIONs. At the same time, fresh-frozen sections were made, and confocal laser scanning microscopy (CLSM, FV3000, Olympus Corporation, Tokyo, Japan) analysis were performed to determine the neuronal affinity of SPIONs. Subsequently, ultrathin sections were made, and transmission electron microscopy (TEM, EP 5018/40/Tecnai Spirit Biotwin 120 kV, FEI Czech Republic s.r.o, Holland) were performed to determine the localization and distribution of SPIONs in the sciatic nerve microstructure. Finally, the amount of iron in nerve tissues at different time points after local nerve injection was detected by ICP-AES, and the neuronal affinity of SPIONs was further quantitatively analyzed.

### Preparation of MF device and quantification of magnetic forces

**Fig. 1:**
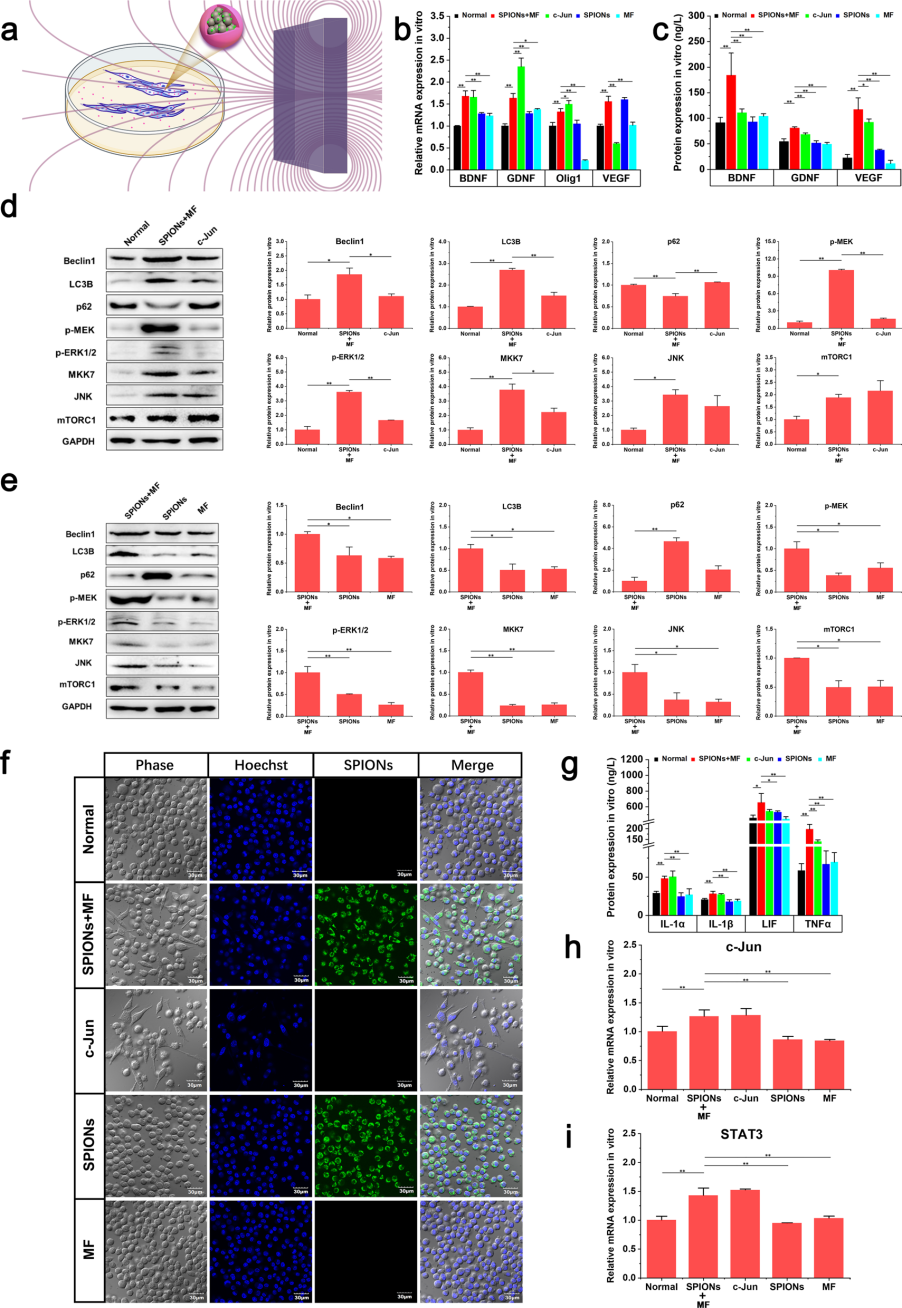
SPION-mediated magnetic actuation induces repair phenotypes in RSC96 cells in vitro.** a** shows the MF environment used in cell experiments in vitro. RSC96 cells coincubated with SPIONs and were magnetized by ingestion of SPIONs. One perpetual cuboid neodymium magnet provided approximately 6.0 T/m of MF to the RSC96 cells at the center of the dish. **b** The relative mRNA expression of regeneration-related neurotrophic factors in different experimental groups was detected through qRT-PCR. **c** The protein expression of regeneration-related neurotrophic factors in different experimental groups was detected by ELISA. **d** The protein expression levels of autophagy markers and regeneration-related signaling pathway biomarkers between the Normal, SPIONs + MF and c-Jun groups were detected through WB. **e** The protein expression levels of autophagy markers and regeneration-related signaling pathway biomarkers between the SPIONs + MF, SPIONs and MF groups were detected through WB. **f** The morphology of RSC96 cells in different experimental groups was observed by confocal laser scanning microscopy. **g** The protein expression of immune-related cytokines in different experimental groups was detected by ELISA. **h**, **i** The relative mRNA expression of Schwann cell repair phenotype-related transcription factors (c-Jun and STAT3) in different experimental groups was detected by qRT-PCR. Each experiment was carried out in triplicate. The relative mRNA expression was calculated by using the 2-ΔΔCT method. The values are represented as the mean ± SD. Scale bar = 30 µm in panel f. *P < 0.05, **P < 0.01

To obtain optimal results, we designed a simple MF generating device for cell experiments in vitro prior to use in vivo based on our previous studies [27, 29]. A 50× 30 × 10 mm perpetual cuboid neodymium magnet (NdFeB N48) was placed on the right side of the ibidi petri dish to expose RSC96 cells to a gradient MF (Fig. 1a).

**Fig. 2:**
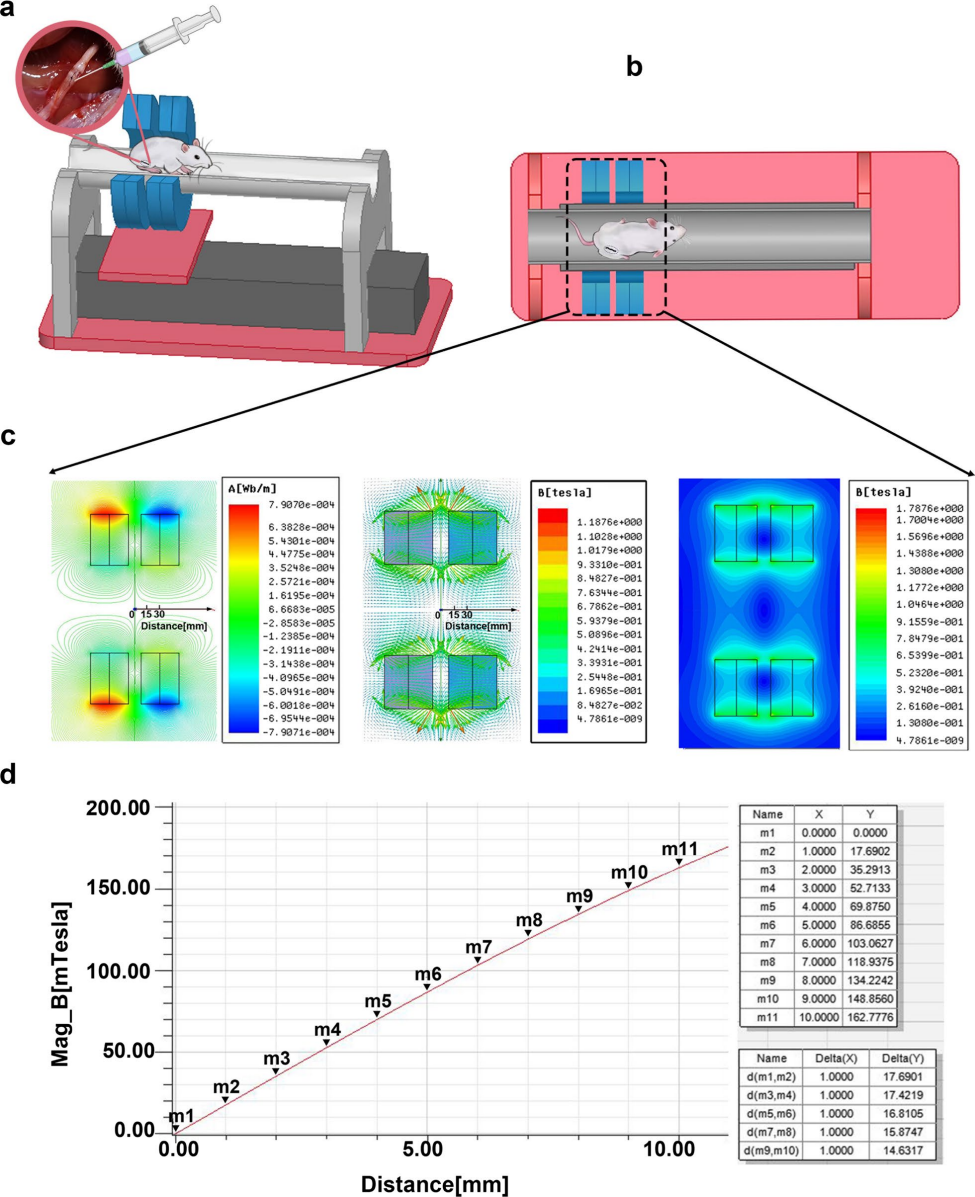
The gradient MF generation device for the in vivo experiment in rats. **a** The MF generating device composed of four circular neodymium magnets. **b** Each two circular magnets form a group, with a 15 mm gap between the two groups. Rats were placed inside circular magnets to expose the sciatic nerve to a gradient MF. **c **There is a large MF gradient inside the circular neodymium magnets. The maximum gradient value appears in the gap between the two groups of circular magnets. **d** A gradient MF of 16.0 T/m is generated in the MF generator

For the in vivo experiment of rats, we designed an MF generating device composed of four circular neodymium magnets (NdFeB N48H) with an inner diameter of 70 mm, an outer diameter of 150 mm and a thickness of 15 mm (Fig. 2a). Each pair of circular magnets form a group, with a 15-mm gap between the two groups. There is a large MF gradient inside the circular neodymium magnets (Fig. 2b). The gradient magnetic field environment was digitally simulated with Comsol Multiphysics 4.3b software (Comsol Multiphysics GmbH, Goettingen, Germany) (Fig. 2c). A digital Gauss meter (Model 425, Lake Shore Cryotronics) was used to measure the magnetic flux density induced by the neodymium magnet setup (Fig. 2d). The rat was placed inside the circular magnets 30 min a day after surgery, and the sciatic nerves were exposed at the center of the gap between the two groups of circular magnets. SPIONs in the sciatic nerve were induced to interact with the gradient MF to produce nano-scale magnetic stimulation of the sciatic nerve.

The exact explanation of the quantification of magnetic forces has been described in our previous studies [27, 29]. A magnetic particle with a magnetic momentum (***m***) can generate magnetic force ***F*** in a magnetic flux density gradient (***∇B***):

1 $${\mathbf{F = (m\cdot\nabla )B}}$$

In our experimental setup, we can measure the value of the gradient magnetic field ***dB/dr***, the density **ρ**, the volume **V** of SPIONs, and the magnetization ***M*** of SPIONs in this field environment. We can assume the net force ***F***_***SPION***_ of each SPION due to a combination of parameters:

2 $${{\varvec{F}}}_{{\varvec{S}}{\varvec{P}}{\varvec{I}}{\varvec{O}}{\varvec{N}}}={\varvec{m}}\frac{{\varvec{d}}{\varvec{B}}}{{\varvec{d}}{\varvec{r}}}={\varvec{\uprho}}\mathbf{V}{\varvec{M}}\frac{{\varvec{d}}{\varvec{B}}}{{\varvec{d}}{\varvec{r}}}$$

The quantity of exogenous iron in sciatic nerve tissue could be measured, from which the number of SPIONs in the nerve (n^SPIONs^_nerve_) could be calculated. The sciatic nerve will thus be subject to a force ***F***_***nerve***_ given by ***F***_***SPION***_ multiplied by the number of SPIONs in the nerve:

3 $$F_{nerve} \, = \,{\mathbf{n}}_{{{\mathbf{nerve}}}}^{{{\mathbf{SPIONs}}}} .{\mathbf{F}}_{{{\mathbf{SPION}}}}$$

### Establishment of rat models of sciatic nerve crush

All works involving animals were in accordance with the National Committee for Science and Technology of Standardized Experimental Animals. Ethical approval for all experiments was granted by the Animal Welfare and Ethical Review Committee of the First Hospital of Jilin University (Approval No. 20210565), and all efforts were made to minimize animal suffering. Eight-week-old Sprague Dawley rats (male, 200–220 g), purchased from Liaoning Changsheng Biotechnology Co., Ltd., were used in all experiments. Rats were deeply anesthetized with an intraperitoneal injection of mixed narcotics (100 mg/kg ketamine plus 10 mg/kg xylazine).

After the rats were fully anesthetized, routine skin preparation and disinfection of the operative field on the lateral aspect of the right thigh were performed. The long sciatic nerve was exposed between the gluteus maximus and quadriceps muscles through a 2 cm long posterolateral longitudinal straight incision on the right thigh. For the nerve crush operation, the sciatic nerve was crushed using a pair of delicate forceps (Fine Science Instruments) two times (30 s each, at 10 s intervals) at the same site 10 mm above the bifurcation into the tibial and common peroneal nerves to create a 2 mm-wide crush lesion. A single 10/0 nylon suture (Mononylon, Ethicon) was passed through the epineurium at the point corresponding to the crushed site to facilitate its identification in the subsequent procedures. For SPION injection, 20 µL SPION solution (300 µg/mL) was slowly injected under the epineurium with a 20 µL Hamilton microsyringe at the distal end of the sciatic nerve crush site. All procedures followed a standard microsurgery technique under a stereomicroscope (Leica Microsystems, Wetzlar, Germany).

Animals were divided into five experimental groups: (1) the normal control group (labeled the ‘Normal’ group and consisting of normal rats without any treatment), (2) the magnetic actuation group (labeled the ‘Crush + SPIONs + MF’ group and consisting of rats that underwent sciatic nerve crush and were then treated with both SPIONs and an MF), (3) the SPIONs control group (labeled the ‘Crush + SPIONs’ group and consisting of animals that underwent sciatic nerve crush and SPION administration but no MF exposure), (4) the MF control group (labeled the ‘Crush + MF’ group and consisting of animals that underwent sciatic nerve crush and MF exposure but did not receive SPIONs), (5) and the surgical control group (labeled the ‘Crush’ group and consisting of animals that underwent only sciatic nerve crush and did not receive SPIONs or MF exposure).

All animals undergoing surgery were given appropriate postoperative analgesia and monitored daily. Animals were housed under a 12/12 h light/dark cycle with free access to food and water. To investigate whether magnetic stimulation can promote recovery after sciatic nerve crush injury, rats in the magnetic actuation group and MF control group were placed in an MF generating device for 30 min every day after surgery and subjected to a daily gradient MF effect. At 3, 7, 14 and 21 days postoperatively, the histomorphology, motor behavior, electrophysiological function and regeneration-related molecular markers of the sciatic nerve in five experimental groups were measured and analyzed. In this study, to investigate the effect of magnetic actuation on the specific repair phenotypes of denervated Schwann cells after nerve injury, the sciatic nerve tissue segment at the distal end of the crush site was selected and analyzed.

### Fresh-frozen sections and CLSM

The distribution and localization of SPIONs in nerve tissue were observed through frozen sections by using CLSM imaging after sub-epineurial injection at different times. Animals were euthanized 1, 3, 7, and 14 days after sub-epineurial injection, and sciatic nerve tissue containing the SPION injection site and its distal 2 cm was harvested as quickly as possible. The sciatic nerve tissue was placed in a special small box (approximately 3 cm in diameter). The tissue was immersed in optimal cutting temperature (OCT) embedding compound, and the container was held steady and fat while beign placed in a small cup containing liquid nitrogen. After the frozen block is made, it can be put into the freezer slicer. On the sciatic nerve, a continuous cross section was made from the injection site to the distal end with a thickness of 10 μm. Nuclei were stained with DAPI (4,6-diamino-2-phenyl indol, Ex/Em: 405/430–470 nm). The localization and persistence of SPIONs (Ex/Em: 488/500–580 nm) in the nerve was analyzed by CLSM. The images were captured with a 60 × oil immersion objective at 3.2 × magnification. Subsequently, as an additional validation experiment, TEM was used to directly observe the presence and localization of the SPIONs in the nerve ultrastructure.

### Optical microscopy and TEM analysis

Semithin and ultrathin sections with optical microscopy and TEM observations were used for the quantitative morphological analysis of sciatic nerve regeneration. Distal sciatic nerves to the crush site at 3, 7, 14 and 21 days after injury were dissected and postfixed with 4% PFA and 3% glutaraldehyde in 0.1 m phosphate buffer. Nerves were osmicated with 1.5% osmium tetroxide for 90 min, dehydrated and embedded in epoxy resin. A series of 5-µm-thick semithin transverse Sections 10 mm distal to the lesion were cut using a PowerTome-XL ultramicrotome (RMC, USA) and stained with 1% toluidine blue for 2–3 min for optical microscopy examination (IX51, Olympus Corporation, Tokyo, Japan). All semithin sections were observed and photographed at 200 × and 1000 × magnification. Ultrathin Sections (100 nm) were cut immediately after the series of semithin sections by means of the same ultramicrotome and mounted onto 100 mesh Cu grids coated with formvar. The sections were stained with saturated aqueous solution of uranyl acetate and lead citrate. Pictures were observed using TEM operating at 120 kV and then analyzed using image analysis software (Image-Pro Express, version 6.0.0.319, Media Cybernetics, Silver Springs, MD, USA). The type and number of various nerve fibers as well as the myelin sheath thickness and G-ratio (axon/fiber diameter ratio) of myelinated nerve fibers can be accurately identified and measured. In all the animals, the nerves were crushed at the same site and then sliced at the same location from the crush point.

### Immunofluorescent staining of sciatic nerve

At the described timepoints (3rd, 7th, 14th and 21st days) following surgery, nerves were dissected, rinsed and fixed in 4% PFA for 5 h at 4 °C. Then, the sciatic nerves were sectioned using a paraffin section system. Nerve sections were permeabilized with 0.2% Triton X-100 for another 15 min at room temperature and blocked for 1 h in 10% normal goat serum (1:50, DAKO, USA) at room temperature. The sciatic nerve sections were stained for double immunofluorescence using mouse anti-neurofilament heavy chain antibody (1:500, Santa, sc-32729) and rabbit anti-S100β antibody (1:100, Abcam, Ab52642). Nerve sections were coincubated with primary antibodies overnight at 4 °C, followed by incubation with goat anti-mouse immunoglobulin G (IgG) secondary antibody with Alexa Fluor 488 conjugate (1:100; Abcam) and goat anti-rabbit IgG secondary antibody with Alexa Fluor 594 conjugate (1:100; Abcam) for 2 h at room temperature. Nuclei were stained with DAPI (Ex/Em: 405/430–470 nm). All procedures were accompanied by rinsing three times in PBS for 5 min each. Finally, the slides were photographed with CLSM to obtain the nerve microstructure of the sciatic nerve during regeneration.

### Sciatic functional index (SFI) analysis

For the SFI analysis, animals were tested in a confined walkway measuring 60 cm long and 10 cm wide, with a dark shelter at the end. White paper was placed on the floor of the rat walking corridor. Prior to any surgical procedure, all rats were trained to walk in the corridor. The rats were held by the back, and their hind feet were pressed down onto a stamp pad soaked with water-soluble black ink. The animals were immediately allowed to walk along the corridor, leaving their footprints on the paper. The tracks left by the walking animals were recorded at days 3, 7, 14 and 21 postoperatively.

The tracks were evaluated for three different parameters: (1) distance from the heel to the third toe, the print length (PL); (2) distance from the first to the fifth toe, the toe spread (TS); and (3) distance from the second to the fourth toe, the intermediary toe spread (ITS). All three measurements were taken from the experimental (E) and normal (N) sides. Using the following formula derived by Bain et al., the SFI is calculated as follows [30]:

4 $${\mathbf{SFI}} = - {38.3}\times \left( {{\mathbf{EPL}} - {\mathbf{NPL}}} \right)/{\mathbf{NPL}} + {109.5} \times \left( {{\mathbf{ETS}} - {\mathbf{NTS}}} \right)/{\mathbf{NTS}} + {13.3} \times \left( {{\mathbf{EIT}} - {\mathbf{NIT}}} \right)/{\mathbf{NIT}} - {8.8}$$

The walking track analysis clearly showed that rat footprint measurements could reflect the muscle functional status of the hind limbs [31]. The PL is dependent on posterior tibial nerve function through gastrocnemius activation, whereas TS and ITS reflect common peroneal nerve innervation of the extensor and intrinsic muscle of the foot. In response to a sciatic nerve lesion, the footprints characteristically demonstrate an increased PL and decreased TS and ITS. The SFI is usually negative after nerve injury, and a higher SFI indicates better function of the sciatic nerve. An SFI score oscillating around 0 is considered normal, whereas an index of -100 indicates total impairment.

### Electrophysiological analysis of the sciatic nerve

At the end of the survival period, electroneuromyography (ENMG) evaluation was performed under general anesthesia and was carried out with a Neuromatic 2000 M/C Neuro-Myograph (Dantec Elektronic Medicinsk Og Videnskabeligt Maleudstyr A/S, Skovlunde, Denmark). Set up an electrical stimulator. Tape a pair of acupuncture needles (0.25 × 25 mm) with a negligible impedance [< 1 Ω]) and 3 mm between them to create electrodes for stimulation. The stimulator and the electrode were connected to a data acquisition unit to take the incoming signals and convert them into digital signals that could be processed with computer software. The sciatic nerves were exposed on both sides under the surgical stereomicroscope as described previously. The ground needle was inserted in the quadriceps femoris muscle of the hindlimb to connect the signal ground plug. Starting with the right hindlimb and placing the recording electrode into the triceps calf, the reference electrode in the Achilles tendon, and the stimulation electrode proximal to the crush lesion site in the sciatic nerves were used. Moisten these electrodes with saline. A stimulation amplitude of 10 mV was chosen, and the compound muscle action potentials (CMAPs) were recorded. The sciatic nerve was stimulated proximal and distal to the crush site twice through stimulation electrodes twice, proximal at the level of the sciatic notch, and distal at the level of the popliteal fossa. The latency of the evoked muscle action potentials was recorded, and the prolongation of latency between two stimuli was calculated. Finally, the distance between the two sets of stimulation electrodes was accurately measured on the sciatic nerve, and the motor nerve conduction velocity (MNCV) was calculated. Both experimental (right) and normal (left) nerves were measured.

### Quantitative real-time PCR (qRT-PCR) analysis

Cell or sciatic nerve samples from different groups were washed three times with precooled PBS. After freezing and grinding, 1 mL of TRIzol was added to every 50 mg of tissue, and total RNA of RSC96 cells and sciatic nerve tissues was extracted. The detailed protocol and primer sequence information of qRT-PCR are presented in the Additional file 1. The relative mRNA expression in different groups was calculated by using the 2-ΔΔCT method. Each reaction was performed three times.

### Western blot (WB) analysis

Cell or sciatic nerve samples from different groups were washed with PBS and subsequently resuspended in radioimmunoprecipitation assay (RIPA, Beyotime, Shanghai, China) lysis buffer. Tissue lysates were then collected by centrifugation (12,000 rpm for 15 min at 4 °C). Protein concentration was determined using a bicinchoninic acid (BCA, Thermo Scientific, California, USA) protein assay kit. Proteins were separated by 10% SDS polyacrylamide gel electrophoresis (SDS-PAGE), followed by transfer onto polyvinylidene difluoride (PVDF) membranes (Millipore, Bedford, MA, USA). Then, the PVDF membranes were blocked with 5% nonfat milk in Tris-buffered saline solution for 1 h. Subsequently, the membranes were incubated overnight at 4 °C with the following primary antibodies: rabbit monoclonal anti-Beclin1 (1:1000, Abcam, catalog number ab207612, Cambridge, UK), rabbit polyclonal anti-LC3B (microtubule associated protein1 light chain 3, 1:2000, Abcam, catalog number ab48394, Cambridge, UK), rabbit polyclonal anti-p62 (1:50,000, Cell Signaling technology, catalog number 5114S, Danvers, MA, USA), rabbit monoclonal anti-p-MEK (1:5000, Abcam, catalog number ab96379, Cambridge, UK), rabbit polyclonal anti-p-ERK1/2 (1:1000, Wanleibio, catalog number WLP1512, Shenyang, China), rabbit monoclonal anti-MKK7 (1:1000, Abcam, catalog number ab239843, Cambridge, UK), rabbit monoclonal anti-JNK (1:10,000, Abcam, catalog number ab124956, Cambridge, UK), rabbit monoclonal anti-mTORC1 (mammalian target of rapamycin complex-1, 1:5000, Abcam, catalog number ab92477, Cambridge, UK), rabbit monoclonal anti-N-cadherin (1:20,000, Abcam, catalog number ab76011, Cambridge, UK), rabbit monoclonal anti-NCAM (neural cell adhesion molecules, 1:1000, Abcam, catalog number ab220360, Cambridge, UK), rabbit polyclonal anti-MBP (myelin basic protein, 1:6000, Proteintech, catalog number 10458–1-AP, Rosemont, USA), rabbit polyclonal anti-Periaxin (1:2000, Boster, catalog number A01686, Pleasanton, USA) and mouse monoclonal anti-GAPDH (Proteintech, 1:1000). After three washes in PBST buffer, membranes were incubated at 37 °C for 1 h with the secondary antibodies horseradish peroxidase (HRP)-labeled goat anti-rabbit IgG (H + L) (1:1000, catalog number A0208, Beyotime Biotechnology, Shanghai, China,) and HRP-labeled goat anti-mouse IgG (H + L) antibodies (1:1000, catalog number A0216, Beyotime Biotechnology, Shanghai, China) and then washed three times in PBST. The special bands were visualized using an electrochemiluminescence (ECL) method (Millipore, Bedford, MA, USA). Tanon image analysis software (Tanon Science & Technology, Shanghai, China) was used to conduct grayscale analysis for protein expression. Experiments were carried out in triplicate.

### ELISA analysis

Total proteins from different groups were extracted by RIPA lysis buffer, and the protein content was quantified by the BCA method as previously described. Protein concentrations of interleukin-1α (IL-1α), interleukin-1β (IL-1β), leukaemia inhibitory factor (LIF), tumor necrosis factor-alpha (TNFα), brain-derived neurotrophic factor (BDNF), glial cell line-derived neurotrophic factor (GDNF), oligodendrocyte transcription factor 1 (Olig1) and vascular endothelial growth factor (VEGF) were detected using an ELISA kit (Huyu Biological Technology, Shanghai, China). Since the concentration of target protein is directly proportional to the absorbance at 450 nm, the concentration of target protein can be calculated by measuring the absorbance value at 450 nm by ELISA. The detailed protocol of ELISA is presented in the Additional file 1.

### Immunohistochemistry (IHC) analysis

Specimens were fixed with 4% PFA for 5 h and embedded in paraffin. Prior to immunohistochemistry, nerve sections were dewaxed and rehydrated in PBS (pH 7.4). Then, the nerve sections were incubated with 0.6% hydrogen peroxide for 30 min. To block nonspecific immunoreactions, the sections were incubated with 10% normal goat serum (1:50, DAKO, USA). Subsequently, sections were incubated with primary antibodies overnight at 4 °C. According to the parameters to be tested, the following primary antibodies were chosen: rabbit polyclonal anti-BDNF (1:400, Boster Biological Technology Co., Ltd, catalog number PB9075, California, USA), rabbit polyclonal anti-GDNF (1:400, Boster Biological Technology Co., Ltd, catalog number PA1465, California, USA), rabbit polyclonal anti-Olig1 (1:400, Beijing Biosynthesis Biotechnology Co., Ltd. catalog number bs-8548R, Beijing, China), rabbit polyclonal anti-VEGF (1:500, Proteintech Group, Inc, catalog number 19003–1-AP, Rosemont, USA), rabbit polyclonal anti-MBP (1:500, Proteintech Group, Inc, catalog number 10458–1-AP, Rosemont, USA) and rabbit polyclonal anti-periaxin (PRX) antibodies (1:400, Boster Biological Technology Co., Ltd, catalog number A01686, California, USA). They were washed three times with PBS and incubated in biotinylated goat anti-rabbit IgG solution for 1 h at 37 °C. HRP-labeled secondary antibodies were applied for 1 h. Then, all sections were incubated with 3,3'-diaminobenzidine tetrahydrochloride chromogenic substrate solution (DAB, DAKO, USA) for 10 min. The immunohistochemistry results were assessed under an optical microscope.

### Statistical analysis

The normality of all data distributions was checked with the Kolmogorov–Smirnov test. Continuous variables presenting a normal distribution, such as quantitative data of protein detected by WB and ELISA, immunohistochemical protein quantification data, qRT-PCR relative gene expression data, amount of iron in the tissues, SFI, CMAPs, and serum biochemical parameters, were represented as the mean ± standard deviation (SD) and compared between groups by one-way analysis of variance (one-way ANOVA). When the presence of significant changes was observed, post hoc multiple pairwise comparisons were carried out using the Student-Neuman-Keuls (SNK) test.

The distributions of the G-ratio of myelinated nerve fibers and the MNCV were found to be nonnormal by the Kolmogorov–Smirnov test; therefore, their values were represented by the median and interquartile range (Q1–Q3). Due to violation of the normality assumption, the G-ratio and MNCV results were analyzed by the nonparametric Friedman test, followed by pairwise comparisons using the Wilcoxon signed-rank test.

All of the analyses were conducted with SPSS (version 18.0, Chicago, IL, USA), and *P* < 0.05 was considered to be statistically significant.

## Results and discussion

### Synthesis and characterization of fluorescent-magnetic SPIONs

**Fig. 3:**
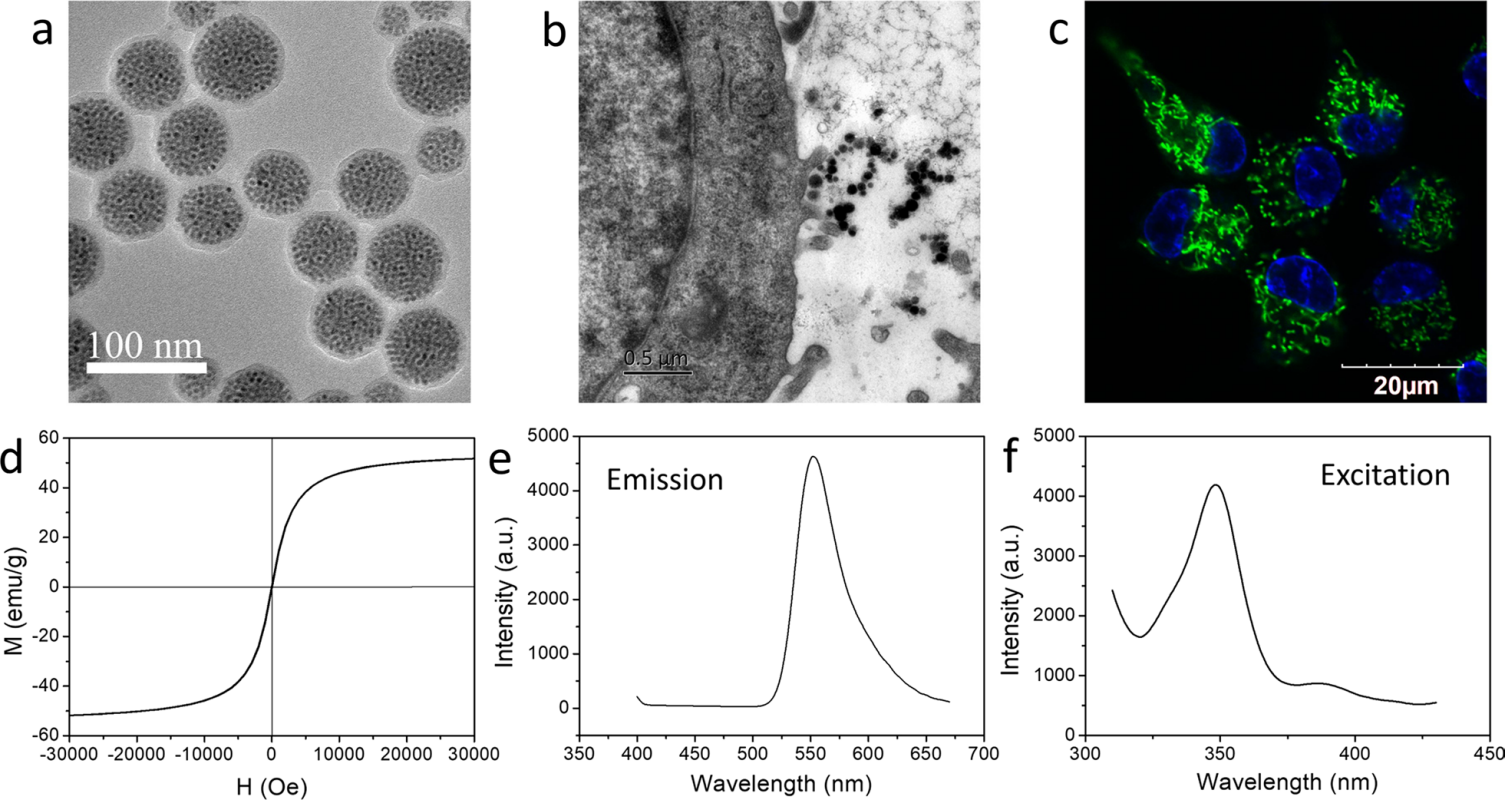
Synthesis and characterization of fluorescent-magnetic bifunctional SPIONs. **a** The high resolution transmission electron microscopy (HRTEM) image shows that SPIONs have an uniform size distribution, with a Fe_3_O_4_·Rhodamine 6G SPs core size of 50 nm and a thin PDA shell of about 6 nm. **b** TEM image shows the successive stage of SPIONs internalization, which suggest that SPIONs has excellent biocompatibility and neuronal affinity. **c** CLSM image of RSC96 cells after incubation with SPIONs exhibit a strong green fluorescence, which demonstrates that the SPIONs possess an ideal capacity to magnetize Schwann cells. **d** Magnetic hysteresis curve of SPIONs shows the saturation magnetization (M_s_) is 51 Am^2^/kg and without any evident remanence or coercivity, suggesting the superparamagnetic property of SPIONs. Photoluminescence spectra of SPIONs show optimal emission **(e)** and excitation **(f)** wavelengths at around 556 and 350 nm

Magnetic actuation relies on two main procedures: magnetizing the therapeutic cells with magnetic particles and applying an MF over the target body region to stimulate the magnetized therapeutic cells. Of all magnetic particles that are available, iron oxide NPs are the most practical choice for magnetic actuation for a number of reasons, including a high magnetic moment, availability, biocompatibility and low toxicity [32, 33]. In this study, we designed and prepared novel fluorescent-magnetic bifunctional SPIONs for neural regeneration therapeutics. The SPIONs are fabricated by coating preassembled Fe_3_O_4_·Rhodamine 6G SPs core with PDA shell (Fig. 3a). This kind of SPIONs possess the following characteristics. (1) Due to the polymerization effect of Fe_3_O_4_ NPs, SPIONs have good superparamagnetism and higher magnetization efficiency. The as-prepared SPIONs possess ideal superparamagnetic properties, since they are assembled by Fe_3_O_4_ NPs with a size of 5.8 nm (Fig. 3d). It is known that magnetic NPs including Fe_3_O_4_ or γ-Fe_2_O_3_ will transfer from the ferromagnetic state to the superparamagnetic state when their diameters reduce to less than 20 nm [34, 35]. Different from individual Fe_3_O_4_ NPs, the assembled superparticle structure greatly accelerates the accumulation of Fe_3_O_4_ NPs in the cells, which is beneficial for providing a stronger mechanical force to manipulate the cells. (2) In addition, because dopamine (DA) is one of the most important neurotransmitters widely secreted in organisms, the PDA shell endows our SPIONs with excellent biocompatibility, colloid stability, physical stability and neuronal affinity (Fig. 3b, c). (3) Finally, Rhodamine 6G, as a fluorescent molecule, endows the SPIONs with excellent fluorescence characteristics, exhibit a strong green fluorescence (Fig. 3c) emission at around 556 nm (Fig. 3e) under 350 nm excitation (Fig. 3f), enabling real-time observation of the interaction between the SPIONs and the target cells.

### SPION-mediated magnetic actuation induces repair phenotypes of RSC96 cells in vitro

To prove that SPIONs can be exploited to induce repair phenotypes of Schwann cells in vitro under an external MF, experiments were carried out inside a constant magnetic flux density gradient generated by one perpetual cuboid neodymium magnet, which provided an MF of approximately 6.0 T/m to the RSC96 cells at the center of the dish (Fig. 1a). According to our characterization results of magnetic properties of SPION (Fig. 3d), the saturation magnetization (***M***_***s***_) of SPION is 51 Am^2^/kg and the magnetization (***M***) value of SPION in this MF is 14 Am^2^*/*kg. According to Eq. (2), a single SPION can generate a force of approximately 0.26 × 10^–4^ pico-Newton (pN). After incubation with the SPIONs at a concentration of 15 μg/mL, the number of SPIONs taken up by the RSC96 cells reached 2.59 ± 0.15 × 10^4^ SPIONs per cell (The detailed method used to determine the number of SPIONs taken by cells is presented in Additional file 1). Thus, according to **Eq. (****3****)**, it can be deduced that each RSC96 cell experiences approximately 0.67 pN magnetic stimulation. Through in vitro experiments with RSC96 cells, we demonstrated that magnetic stimulation mediated by SPIONs can induce a series of phenotypic changes in RSC96 cells, which is consistent with the typical characteristics of repair Schwann cells. These characteristics include a number of components, all of which support repair: (1) upregulation of proteins that support neuronal survival and promote axonal regeneration, (2) activation of an innate immune response, (3) structural reorganization, and (4) activation of myelinophagy [5, 15].

#### SPION-mediated magnetic actuation upregulates the expression of neurotrophic factors in Schwann cells

The expression of neurotrophins, including BDNF, GDNF, Olig 1 and VEGF, in different experimental groups was detected by qRT-PCR. In the SPIONs + MF group, RSC96 cells were treated with magnetic stimulation mediated by SPIONs for 24 h. As shown in Fig. 1b, the mRNA expression levels of BDNF, GDNF, Olig1 and VEGF in RSC96 cells in the SPIONs + MF and c-Jun groups were significantly higher than those in the Normal, SPIONs and MF control groups.

To verify the qRT-PCR results, we used ELISA to detect the protein expression levels of neurotrophins in the cell culture medium of different experimental groups. As shown in Fig. 1c, the ELISA results were consistent with the qRT-PCR results. The protein expression levels of BDNF, GDNF and VEGF in the SPIONs + MF group were significantly higher than those in the Normal, c-Jun (positive control), SPIONs and MF groups.

These results suggested that SPION-mediated magnetic actuation can upregulate the expression of RSC96 neurotrophins, which is similar to JNK agonists, while neither SPIONs nor MF treatment alone increased the expression of these proteins.

#### SPION-mediated magnetic actuation activates autophagy in Schwann cells

In the early stage of peripheral nerve injury, the axon ruptured, and Wallerian degeneration occurred at the distal end of the injured nerve. In the first step of Wallerian degeneration after injury, autophagy of repair Schwann cells is strongly activated, and the redundant myelin sheath is removed, creating a favorable microenvironment for the extension and growth of new regenerative axons. Thus, repair Schwann cells play a major role in neural regeneration by breaking down their own redundant myelin fragments by activating autophagy. Activation of autophagy is considered a marker of the conversion of Schwann cells to repair cells.

We detected the protein expression levels of autophagy-related biomarkers, such as Beclin1, LC3B, and p62, in RSC96 cells by WB. As shown in Fig. 1d, Beclin1 and LC3B protein levels in the SPIONs + MF group were significantly higher than those of the Normal and c-Jun groups. The p62 protein is an important substrate in autophagy, and its content is inversely proportional to autophagy activity. Therefore, the expression trend of p62 was opposite to that of Beclin1 and LC3B, and the protein content of p62 in the SPIONs + MF group was significantly lower than those in the Normal and c-Jun groups. These results suggested that autophagy in RSC96 cells was activated by SPION-mediated magnetic stimulation.

To further investigate whether SPIONs or MF alone could induce similar autophagy responses in RSC96 cells, we compared the expression levels of these autophagy biomarkers between the SPIONs + MF, SPIONs and MF groups. The results in Fig. 1e show that the protein expression of Beclin1 and LC3B in the SPIONs + MF group was significantly increased compared with those in the SPIONs and MF groups, while the p62 protein showed the opposite trend. These results suggested that magnetic actuation generated by the interaction of SPIONs with external gradient MF, rather than SPIONs or MF alone, is the key initiator of autophagy activation in RSC96 cells.

#### SPION-mediated magnetic actuation induces Schwann cell elongation and branching

Another key function of repair Schwann cells is to guide growing axons to their target organs by forming regeneration tracks known as bands of Bungner [36]. Repair-supportive Schwann cells elongate and branch, which allow cells to overlap and connect with each other and promote the formation of compact cellular columns (bands of Bungner). These results in a distinctive repair cell morphology that is necessary for the formation of regeneration trajectories for regenerating axons [14].

The cultures from the positive control group (the c-Jun group) were recorded with differential interference contrast (DIC) optics and showed a striking elongated bipolar and branched morphology (Fig. 1f). As shown in Additional file 2: Movie S1, we used time-lapse imaging to monitor the effect of SPION-mediated magnetic actuation on the morphology of RSC96 cells. In the SPIONs + MF group, similar elongation and branching cell morphology was observed as in the c-Jun group, which was very different from that of RSC96 cells in the Normal, SPIONs and MF groups. These results suggested that magnetic actuation mediated by SPIONs could induce structural reorganization in RSC96 cells that is favorable for the formation of bands of Bungner, which are essential for nerve repair.

#### SPION-mediated magnetic actuation upregulates the expression of immune-related cytokines in Schwann cells

Another important feature of repair Schwann cells compared to other lineages of Schwann cells after nerve injury is activation of an innate immune response, comprising the upregulation of cytokines including IL-1α, IL-1β, LIF, and TNFα [37, 38]. These cytokines recruit macrophages, which help Schwann cells clear redundant myelin and stimulate the formation of blood vessels [39–42]. The protein levels of immune-related cytokines (IL-1α, IL-1β, LIF and TNFα) in the cell culture medium of different experimental groups were determined by ELISA. The results in Fig. 1g show that the protein expression levels of these cytokines were the highest in the SPIONs + MF and c-Jun groups and were significantly higher than those in the Normal, SPIONs and MF groups.

#### SPION-mediated magnetic actuation upregulates the expression of transcription factors associated with repair phenotypes in Schwann cells

It is now recognized that the activation of a transcriptional repair program in Schwann cells is a critical determinant of the execution of the Schwann cell injury response and success of axon regeneration [5, 43, 44]. This repair program is controlled transcriptionally by mechanisms involving the transcription factors c-Jun and STAT3 (signal transducer and activator of transcription-3). These transcription factors are rapidly upregulated in Schwann cells after injury and are essential for the generation and maintenance of repair Schwann cells.

As shown in Fig. 1h, i, c-Jun and STAT3 were significantly upregulated by a specific JNK agonist (anisomycin) in the positive control group (c-Jun). In the magnetic actuation group (SPIONs + MF), magnetic stimulation was applied to RSC96 cells by SPIONs and a gradient MF, resulting in significant upregulation of c-Jun and STAT3 expression. This pattern of upregulation was consistent with the positive control group and significantly higher than other normal, SPIONs, and MF controls, confirming the hypothesis that magnetic actuation mediated by SPIONs can act as an effective mechanical intervention to induce repair phenotypes in Schwann cells by upregulating the expression of c-Jun and STAT3.

#### SPION-mediated magnetic actuation activates signaling pathways associated with repair phenotypes in Schwann cells

At present, multiple signaling pathways are known to be involved in Schwann cell repair phenotypes after nerve injury and carry out distinct functions [45]. The Raf-MEK-ERK1/2 pathway is particularly implicated in cytokine expression, macrophage recruitment and myelin breakdown. ERK1/2 phosphorylation is rapidly induced in repair Schwann cells after injury and implicated in the upregulation of the major macrophage recruitment signal monocyte chemoattractant protein-1 (MCP-1) [46, 47]. The Rac1-MKK7-JNK pathway activates c-Jun and regeneration-related genes and promotes myelinophagy. The mTORC1 pathway is activated in Schwann cells by injury. This is required for c-Jun activation because TORC1 promotes c-Jun translation. Genetic inactivation of the TORC1 pathway results in subdued activation of c-Jun and other repair cell genes.

We used WB to detect the expression of major biomarkers in the above signaling pathways. As shown in Fig. 1d, e, the protein expression of p-MEK and p-ERK1/2 in RSC96 cells was significantly increased in the magnetic actuation group compared with the control groups (normal, positive, SPIONs, and MF control groups), indicating that the Raf-MEK-ERK1/2 signaling pathway was activated. The expression of MKK7 and JNK proteins in the magnetic actuation group and positive control group was significantly higher than that in the normal control, SPIONs control and MF control groups. This demonstrated that SPION-mediated magnetic stimulation (as applied to the SPIONs + MF group) had the same biological effect as a JNK agonist (positive control group, c-Jun), both of which can significantly activate the Rac1-MKK7-JNK signaling pathway. Western blotting for mTORC1 protein showed the same trend, the magnetic actuation group and positive control group were also comparable in the expression of mTORC1, which was significantly higher than the control groups (normal control, SPIONs control and MF control groups).

After peripheral nerve injury, denervated Schwann cells convert to repaired Schwann cells, and undergo a radical change in functional status compared with the Schwann cells of uninjured nerve, provide powerful support for regeneration [5, 7–10, 13, 48–53]. Most of the properties of repair Schwann cells that are distinctive and differentiate them from myelin or Remak Schwann cells show a distinct molecular expression profile [5]. From the in vitro experiments, we found that SPIONs can produce pN-level magnetic stimulation of RSC96 cells under an external gradient MF. This magnetic stimulation can actuate a series of gene expression profile changes in RSC96 cells and finally show typical repair support phenotypes.

### In vivo toxicology, pharmacokinetics and neuronal affinity of SPIONs

#### SPIONs possess negligible toxicity and excellent biocompatibility

Before further use of SPIONs for in vivo neural magnetic actuation, we systematically investigated the toxicology and pharmacokinetics of SPIONs in rats to assess the biosafety of SPIONs. SPIONs were suspended in sterilized distilled water (1 mg/mL) and injected via the caudal vein at a dose of 1 mg/kg body (200 µL), and the same volume of sterilized distilled water was injected into the control rats. The general status, body weight trends, blood biochemistry analyses, and histological analysis of rats were monitored during the 14-day postinjection period.

**Fig. 4:**
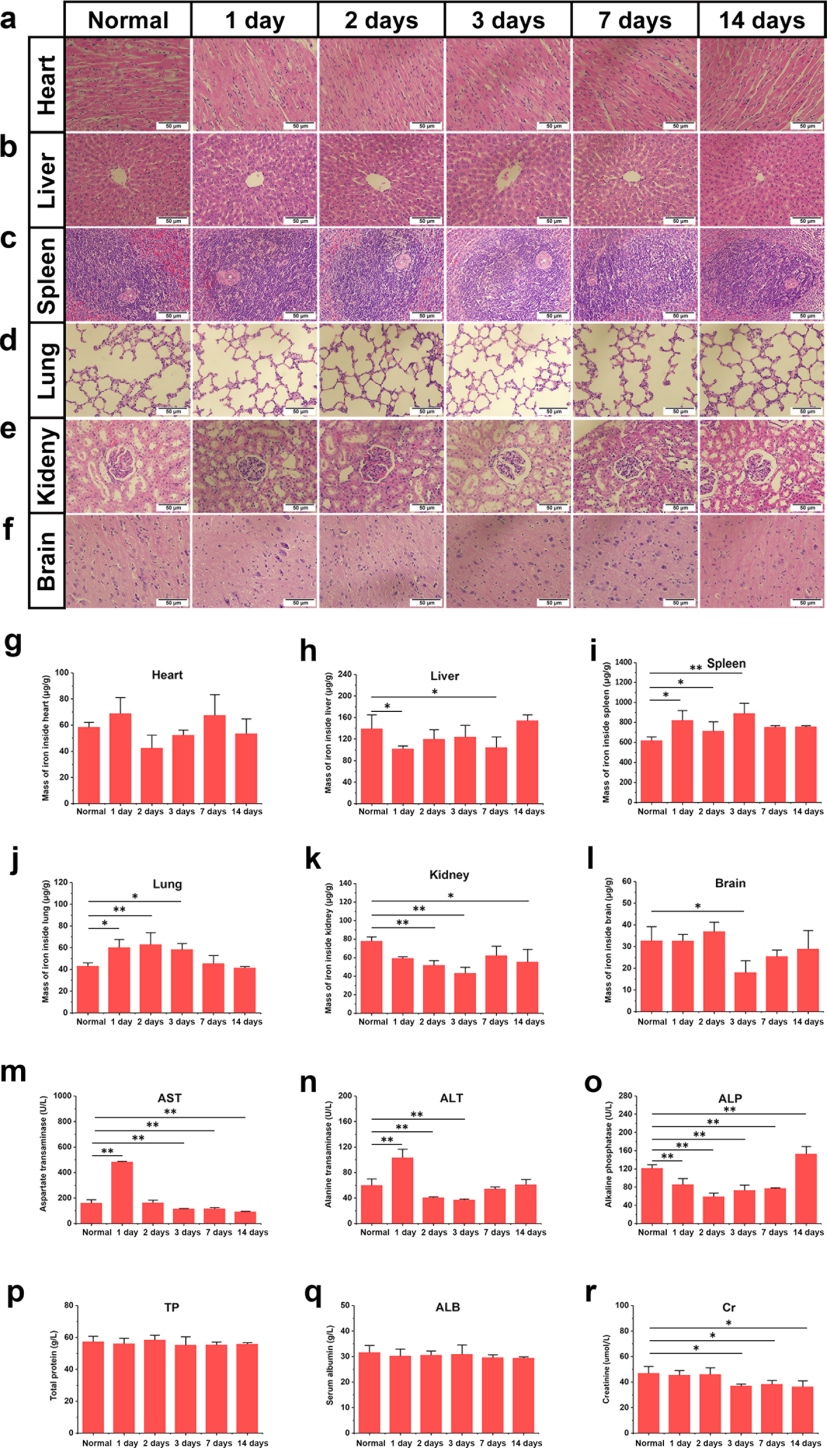
In vivo toxicological studies of SPIONs. Pathological damage in the heart **(a)** liver **(b)** spleen **(c)** lung **(d)** kidney **(e)** and brain (**f)** of normal and SPION-injected rats at 1, 2, 3, 7 and 14 days were detected by histopathological sections and HE staining. **g**–**l** Iron content in these organs of normal and SPION-injected rats at 1, 2, 3, 7 and 14 days were detected by ICP-AES measurement. **m**–**r** The AST, ALT, ALP, TP, ALB, and Cr levels of normal and SPION-injected rats at 1, 2, 3, 7 and 14 days were determined by serum biochemical analysis. Each experiment was carried out in triplicate. The values are represented as the mean ± SD. Scale bar = 50 µm in panels a-f. *P < 0.05, **P < 0.01

Neither death nor a significant body weight drop was observed in the SPION-injected rats within 14 days, and the rats were generally in good condition. The SPION-injected rats were euthanized at 1, 2, 3, 7, and 14 days after injection. Histopathological analysis of the major organs was performed to observe potential tissue inflammation or lesions caused by systemic intravenous administration of SPIONs. In our study, no apparent histopathological abnormalities or lesions were observed in the heart, liver, spleen, lung, kidney, or brain at 1, 2, 3, 7 and 14 days after injection (Fig. 4a–f). Subsequently, a serum biochemistry study was performed, and six important hepatorenal indicators, including AST, ALT, ALP, TP, ALB, and Cr, were measured periodically (Fig. 4m–r). AST and ALT in rats treated with SPIONs increased on the first day after intravenous injection but quickly fell back to the normal range on the second day and then were close to the normal control group during the following observation period (Fig. 4m, n). As shown in Fig. 4o–r, SPIONs injection did not cause significant anomalies in other biochemical indicators, suggesting no liver or renal damage.

To further study the systemic distribution and pharmacokinetics of SPIONs through intravenous administration after the low toxicity of SPIONs was determined, exogenous iron content in main organs was detected by ICP-AES (Fig. 4g–l). The results showed that there was no obvious accumulation or residue of SPIONs in organs during the observation period of 14 days, which also proved the good biocompatibility and biosafety of SPIONs.

#### SPIONs possess excellent neuronal affinity

After confirming the low toxicity and good biosafety of SPIONs, we further studied the neural affinity of SPIONs. A 20-µL volume of SPIONs at a concentration of 300 µg/mL (30% of the concentration used for intravenous administration) was locally injected under the epineurium of the sciatic nerve. At 1, 3, 7, and 14 days after injection, the sciatic nerves were harvested for HE staining, fresh-frozen section, and TEM.

**Fig. 5:**
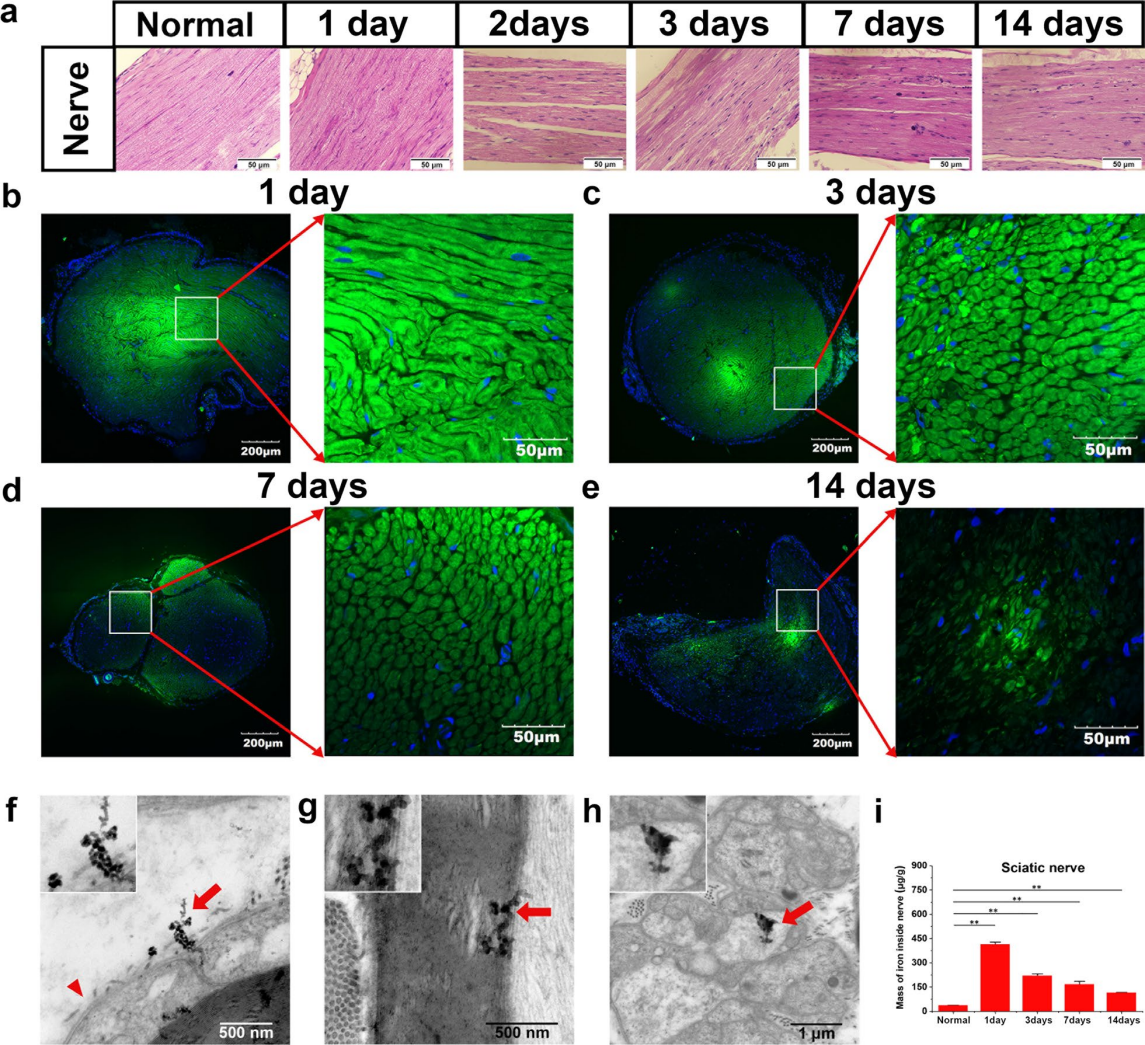
Neurotoxicity and neuronal affinity of SPIONs.** a** HE staining images of nerve tissue sections collected 1, 2, 3, 7, and 14 days after sub-epineurial injection of SPIONs (300 µg/ mL, 20 µL) were used to observe the neurotoxicity of SPIONs. **b**–**e** At 1, 3, 7 and 14 days after sub-epineurial injection at the same concentration, the sciatic nerves were harvested, and freshly frozen sections were obtained. The localization and distribution of SPIONs in the nerve tissue were observed through fluorescence using CLSM. The distribution and localization of SPIONs in nerve ultrastructure were observed directly by high-resolution TEM. **f** SPIONs penetrated the basal membrane of the myelin sheath of Schwann cells and **g** were distributed in the inner myelin lamina of Schwann cells. **h** SPIONs were found in unmyelinated nerve fibers. **i** The iron content of the sciatic nerve at different time points after sub-epineurial injection was quantitatively measured by ICP-AES. Each experiment was carried out in triplicate. The values are represented as the mean ± SD. The long red arrows indicate the SPIONs in panels f–h, and the short red arrowhead indicates the basal membrane of Schwann cells in panel f. *P < 0.05, **P < 0.01

The HE staining images showed no pathological changes, indicating that SPIONs have no neuronal tissue toxicity (Fig. 5a). Fresh-frozen sections of the sciatic nerve were imaged by CLSM. In the cross section of the sciatic nerve, Rhodamine 6G-labeled SPIONs were detected in the axons and myelin sheath of nerve fibers, and a strong green fluorescence signal emitted by SPIONs was observed, which was specifically concentrated in the interior of nerve fibers (Fig. 5b–e). With the passage of time, the fluorescence intensity in the nerve fibers gradually decreased, but 14 days after sub-epineurial injection, SPIONs still showed obvious fluorescence in the nerve fibers (Fig. 5e). These results suggested that SPIONs can be specifically ingested and internalized by nerve fibers.

To further demonstrate the affinity of SPIONs to nerve fibers, TEM was used to directly observe the process of SPION internalization. TEM images showed that SPIONs could penetrate the basal membrane of the myelin sheath of Schwann cells (Fig. 5f) and distribute in the inner myelin lamina (Fig. 5g). SPIONs were also observed in Remak Schwann cells surrounding unmyelinated nerve fibers (Fig. 5h).

Finally, to further quantitatively analyze SPION uptake by the sciatic nerve, ICP-AES measurements were used to measure the iron content of the sciatic nerve at different time points after sub-epineurial injection. The results in Fig. 5i show that the relative weights (W_Fe_/W_tissue_) of iron inside the sciatic nerve were 413.83 ± 14.61 µg/g, 219.21 ± 13.76 µg/g, 167.41 ± 18.10 µg/g and 115.50 ± 2.12 µg/g at 1, 3, 7 and 14 days after sub-epineurial injection, respectively. The iron content in normal sciatic nerve tissue was 36.90 ± 1.00 µg/g. These results indicated that SPIONs have a good affinity for nerve tissue.

### Quantitative calculation of SPION-mediated magnetic force in vivo

Through digital MF simulation, it can be calculated that a gradient MF (***dB/dr***) of 16.0 T/m can be provided inside the MF generating device composed of circular neodymium magnets, and the maximum gradient value appears in the gap between the two groups of circular magnets (Fig. 2). A digital Gauss meter was used to verify the actual magnetic flux density in the MF generator, and the results were in good agreement with the digital simulation results. The magnetization (M) value of SPION in this field is 25 Am^2^/kg according to the magnetic hysteresis curve of Fig. 3d.

According to Fig. 5i, the relative weight (W_Fe_/W_tissue_) of total iron in the sciatic nerve could be detected and quantified at 1, 3, 7, and 14 days after sub-epineurial injection of SPIONs. In contrast, the relative weight (W_Fe_/W_tissue_) of total iron in the normal control group was 36.90 ± 1.00 µg/g. By subtraction, the relative weight of residual exogenous iron in nerve tissue 1, 3, 7, and 14 days after sub-epineurial injection of SPIONs was calculated as 376.93 ± 14.61 µg/g, 182.31 ± 13.76 µg/g, 130.51 ± 18.10 µg/g, and 78.6 ± 2.12 µg/g, respectively. The mean weight of sciatic nerve tissue (W_tissue_) obtained was approximately 0.010 ± 0.006 g, from which we calculated that the absolute weight of residual exogenous iron (W_Fe_) was 3.77 ± 0.14 µg, 1.82 ± 0.14 µg, 1.31 ± 0.18 µg, and 0.79 ± 0.02 µg. The corresponding numbers of SPIONs (n^SPIONs^_nerve_) were 2.04 × 10^10^, 0.98 × 10^10^, 0.71 × 10^10^ and 0.43 × 10^10^. According to Eq. (2), each SPION can generate magnetic forces (*F*_*SPION*_) of approximately 1.24 × 10^–4^ pN in a gradient MF of 16 T/m. According to Eq. (3), magnetic stimulation exerted by SPIONs on the sciatic nerve (*F*_*nerve*_) at 1, 3, 7, and 14 days after sub-epineurial injection was calculated to be 2.53 micro-Newton (µN), 1.22 µN, 0.88 µN, and 0.53 µN, respectively.

SPIONs can be specifically taken up by nerve fibers and Schwann cells after injection through the epineurium, remain stable in the cells and exert a µN-level force on the sciatic nerve under the action of an external gradient MF. These results provide the possibility of using SPION-mediated magnetic actuation to influence the repair phenotypes of Schwann cells in vivo.

### Magnetic actuation mediated by SPIONs promotes nerve regeneration and repair after sciatic nerve crush injury in vivo

After demonstrating that magnetic actuation mediated by SPIONs promoted the transformation of Schwann cells to repair phenotypes in vitro, we evaluated its effect on the generation and maintenance of repair Schwann cells and axonal regeneration in a rat model of peripheral nerve injury. Numerous tests have been devised to assess different aspects of peripheral nerve repair in rats, including axonal regeneration, target reinnervation, and functional recovery. In this experiment, nerve regeneration was evaluated using three commonly employed classes of measures: sciatic histomorphometric assessment, SFI test and sciatic electrophysiological examination.

#### Sciatic histomorphometric assessment

**Fig. 6:**
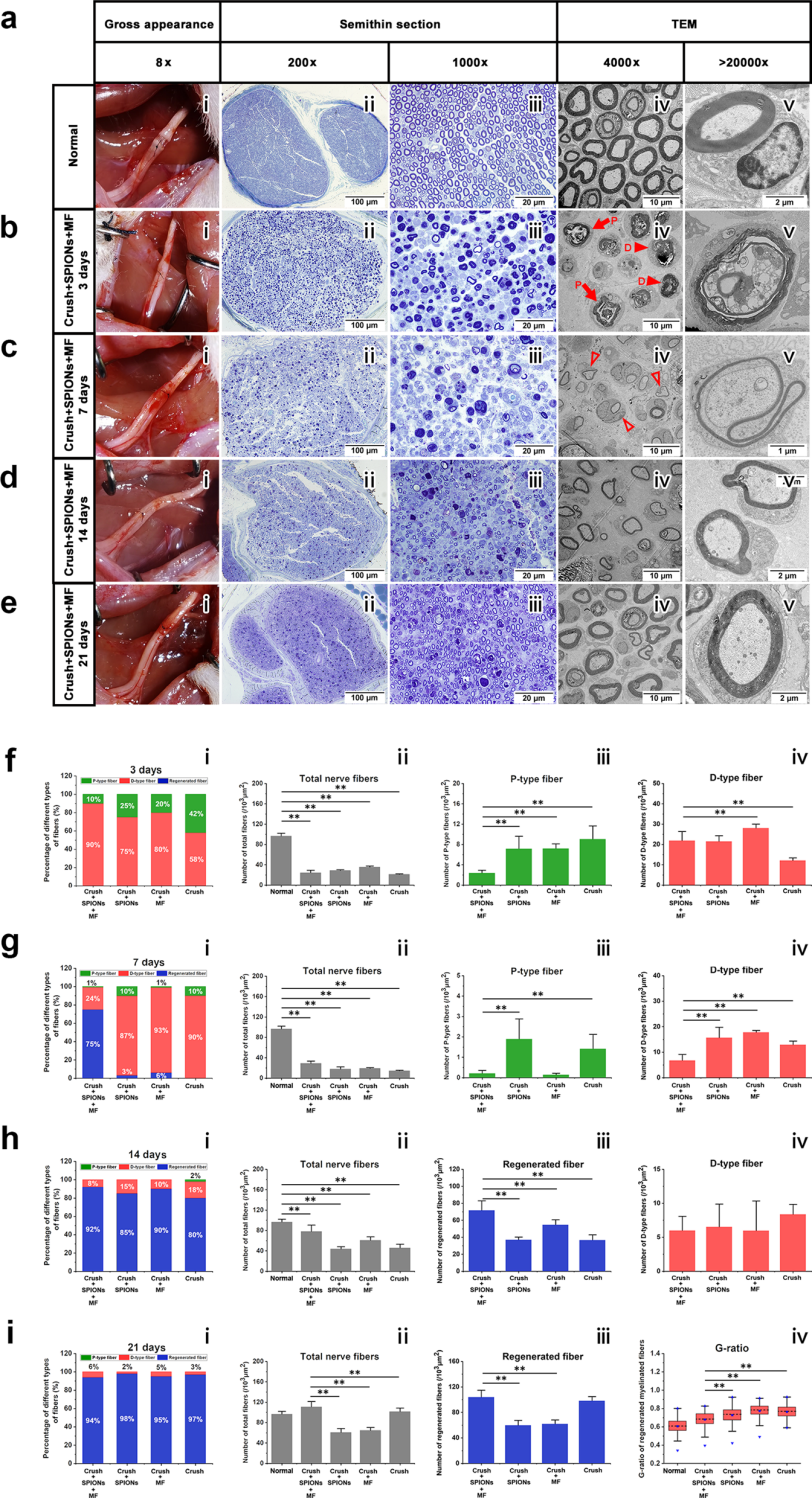
SPION-mediated magnetic actuation promotes morphological regeneration of the sciatic nerve. At 3, 7, 14, and 21 days after sciatic nerve crush injury, the gross appearance, semithin sections and ultrathin sections were observed by stereomicroscopy, optical microscopy, and TEM. The morphology and microstructure of nerves in different experimental groups were observed and quantitatively assessed. **a** The gross appearance (**i**) of the sciatic nerve immediately after crush injury and the microstructure of the normal nerve (**ii**–**v**). **b** In the magnetic actuation (Crush + SPIONs + MF) group, the gross appearance (**i**) and microstructure of the sciatic nerve at 3 days after crush injury were observed and showed obvious Wallerian degeneration (**ii**-**v**). **c** In the Crush + SPIONs + MF group, the gross appearance (**i**) and microstructure of the sciatic nerve (**ii**–**v**) at 7 days after crush injury were observed and showed new regenerated nerve fibers (**iv**, **v**). **d** In the Crush + SPIONs + MF group, the gross appearance (**i**) and microstructure of the sciatic nerve (**ii**-**v**) at 14 days after crush injury were observed, and the thickness of the myelin sheath of the regenerated nerve fibers was increased (**iv**, **v**). **e** In the Crush + SPIONs + MF group, the gross appearance (**i**) and microstructure of the sciatic nerve (**ii**-**v**) at 21 days after crush injury were observed, and the morphology of the regenerated nerve fibers returned to nearly normal (**iv**, **v**). **f** Three days after crush injury, the proportions of various nerve fibers (**i**), the number of total nerve fibers (**ii**), the number of P-type nerve fibers (**iii**), and the number of D-type nerve fibers (**iv**) of the sciatic nerve in the Crush + SPIONs + MF, Crush + SPIONs, MF, and Crush groups were quantitatively assessed. **g** Sciatic nerve morphometric assessment was performed in each of the four experimental groups 7 days after crush injury. **h** At 14 days after crush injury, the proportions of various nerve fibers (**i**), the number of total nerve fibers (**ii**), the number of regenerated nerve fibers (**iii**), and the number of D-type nerve fibers (**iv**) of the sciatic nerve in the four experimental groups were quantitatively assessed. **i** At 21 days after crush injury, the proportions of various nerve fibers (**i**), the number of total nerve fibers (**ii**), the number of regenerated nerve fibers (**iii**) and the G-ratio (**iv**) of the sciatic nerve in the four experimental groups were quantitatively assessed. The long red solid arrows indicate the P-type nerve fibers and the short red solid arrowheads indicate the D-type nerve fibers in panel b (**iv**), the short red hollow arrowheads indicate the regenerated nerve fibers in panel c (**iv**). *P < 0.05, **P < 0.01

According to Fig. 6a(i), immediately after acute compression injury, the crushed areas of all sciatic nerves were flattened, but nerve continuity was preserved. Complete flaccid paralysis of the operative foot was observed following crush injury. All rats survived, with no wound infection or self-mutilation after surgery. At 3, 7, 14, and 21 days after crush injury, the morphological characteristics of regenerated nerves in different experimental groups were evaluated, and histomorphometric analysis of regenerated myelinated nerve fibers was carried out.

Figure 6a(ii–v) shows morphological images of normal sciatic nerve cross sections from semithin sections with optical microscopy and ultrathin sections with TEM. Figure 6b, Additional file 1: Figs. S1a, S2a and S3a show the early stage of Wallerian degeneration at 3 days after sciatic nerve crush injury. Myelin collapsed, and the nerve fibers were divided by myelin into early ovoid structures. This observation provided evidence that crush lesions indeed interrupt the continuity of axons, inducing degeneration of their distal stump. These elliptical structures are known as primary ovoid nerve fibers (P-type fibers) (Fig. 6b(iv), Additional file 1: Figs. S1a(iii, iv), S2a(iii, iv), S3a(iii, iv)), which represent an early demyelinating process. As Wallerian degeneration progresses, P-type fibers are gradually replaced by demyelinating nerve fibers (D-type fibers), which are filled with dense and even folded and curled myelin structures (Fig. 6b(iv, v)), representing the late demyelination process [54]. As shown in Fig. 6f(ii) and b(ii, iii), the total number of nerve fibers decreased significantly in all experimental groups after nerve crush injury compared with the normal control. However, myelin breakdown was faster in the magnetic actuation group, as confirmed by a significant increase in the percentage of D-type fibers in relation to the total number of nerve fibers (Fig. 6f(i); Crush + SPIONs + MF group, 90%, vs. Crush + SPIONs group, 75%, vs. Crush + MF group, 80%, vs. Crush group, 53%). This suggested that magnetic actuation mediated by SPIONs could accelerate the process of Wallerian degeneration and enhance myelin clearance in the early phase of the nerve injury response.

At 7 days after sciatic nerve crush injury, in the advanced stage of Wallerian degeneration, the total number of nerve fibers decreased further (Fig. 6c(ii, iii) and g(ii)) and the proportion of D-type fibers in the total nerve fibers gradually increased (Fig. 6c(iii) and g(i)). The total number of nerve fibers in normal control group was 96.40 ± 5.66 fibers per 1000 µm^2^, decreasing to 24.23 ± 4.94 in magnetic actuation group, 28.72 ± 1.73 in SPIONs control group, 35.47 ± 2.25 in MF control, and 21.08 ± 1.38 in surgical control group (Fig. 6g(ii)). The differences between the experimental groups and the normal control group were significant (*p* < 0.01). The TEM observations (Fig. 6c(iv, v)) added important information to the observations made by optical microscopy (Fig. 6c(ii, iii)) that clear pictures of nerve fiber regeneration could be detected in magnetic actuation nerves. Regenerated nerve fibers were detectable only in the magnetic actuation group (Fig. 6c) and not in the other controls (Additional file 1: Figures S1b, S2b, S3b), which showed the presence of a healthy, smaller caliber and a thinner myelin sheath in comparison to normal nerve fibers (Fig. 6c(iv, v)). These observations demonstrated that magnetic actuation mediated by SPIONs induced the formation of regenerated nerve fibers during the advanced stage of Wallerian degeneration after nerve injury significantly earlier than other controls.

At 14 days after sciatic nerve crush injury, an early stage of nerve regeneration, the total number of nerve fibers increased significantly but was still below normal (Fig. 6d(ii, iii), h(ii)). Clear signs of myelinated nerve fiber regeneration were observed in all groups (Fig. 6d, Additional file 1: Figs. S1c, S2c and S3c), and the number of new regenerated nerve fibers accounted for the majority of all nerve fibers (Fig. 6h(i)). However, the number of regenerated nerve fibers was significantly higher in the magnetic actuation group than in the other controls (Fig. 6h(iii)).

At 21 days after sciatic nerve crush injury, during the middle stage of nerve regeneration, the total number of nerve fibers in all groups had increased to the level of normal nerves (Fig. 6e(ii, iii) and i(ii)), with a further increase in the proportion of new myelinated nerve fibers (Fig. 6i(i, iii)). At the same time, the morphology of regenerated nerve fibers gradually approached normal nerve fibers, the diameter of nerve fibers and the thickness of the myelin sheath increased (Fig. 6e, Additional file 1: Figs. S1d, S2d and S3d), and the G-ratio decreased (Fig. 6i(iv)). Notably, the total number of nerve fibers (Fig. 6i(ii)) and the number of regenerated nerve fibers in the magnetic actuation group (Fig. 6i(iii)) were significantly higher than those in the other controls. The average number of total nerve fibers was 110.70 ± 5.66 fibers per 1000 µm^2^ in the magnetic actuation group, 60.51 ± 7.78 in the SPIONs control group, 64.71 ± 5.77 in the MF control group, and 101.39 ± 7.10 in the surgical control group. The differences between the magnetic actuation group and other control groups were significant (*p* < 0.05). As shown in Fig. 6i(iv), the average G-ratio was 0.69 (0.63–0.74) for the nerve fibers in the magnetic actuation group, while in the SPIONs control, MF control and surgical control groups, it was 0.74 (0.67–0.79), 0.78 (0.74–0.83) and 0.77 (0.72–0.81), respectively. There was no significant difference in the G-ratio between the magnetic actuation group and the normal control group 0.61 (0.56–0.66), but it was significantly lower than that of the above three control groups.

Based on the above morphological analysis of the sciatic nerve, it can be concluded that in the magnetic actuation group, magnetic actuation mediated by SPIONs can promote myelin removal and accelerate the process of Wallerian degeneration in the early stage after nerve injury. During the intermediate stage of Wallerian degeneration, magnetic actuation mediated by SPIONs induces the formation of regenerated nerve fibers and induces more nerve fiber regeneration and better myelin structure in the subsequent stage of regeneration.

#### Regeneration and repair of the sciatic nerve after injury

To observe the process of nerve repair and regeneration, long segmental nerve tissue immunofluorescent staining was used to identify the formation of bands of Bungner and regrowth of axons after nerve crush injury. We performed an immunofluorescent staining protocol on rat sciatic nerve samples 3, 7, 14 and 21 days after crush injury using neurofilament heavy chain antibody and S100β antibody and counterstaining with DAPI dye. In this way, the pattern of axonal regrowth and the interaction between Schwann cells and regenerated axons can be clearly revealed at different time points following injury.

**Fig. 7:**
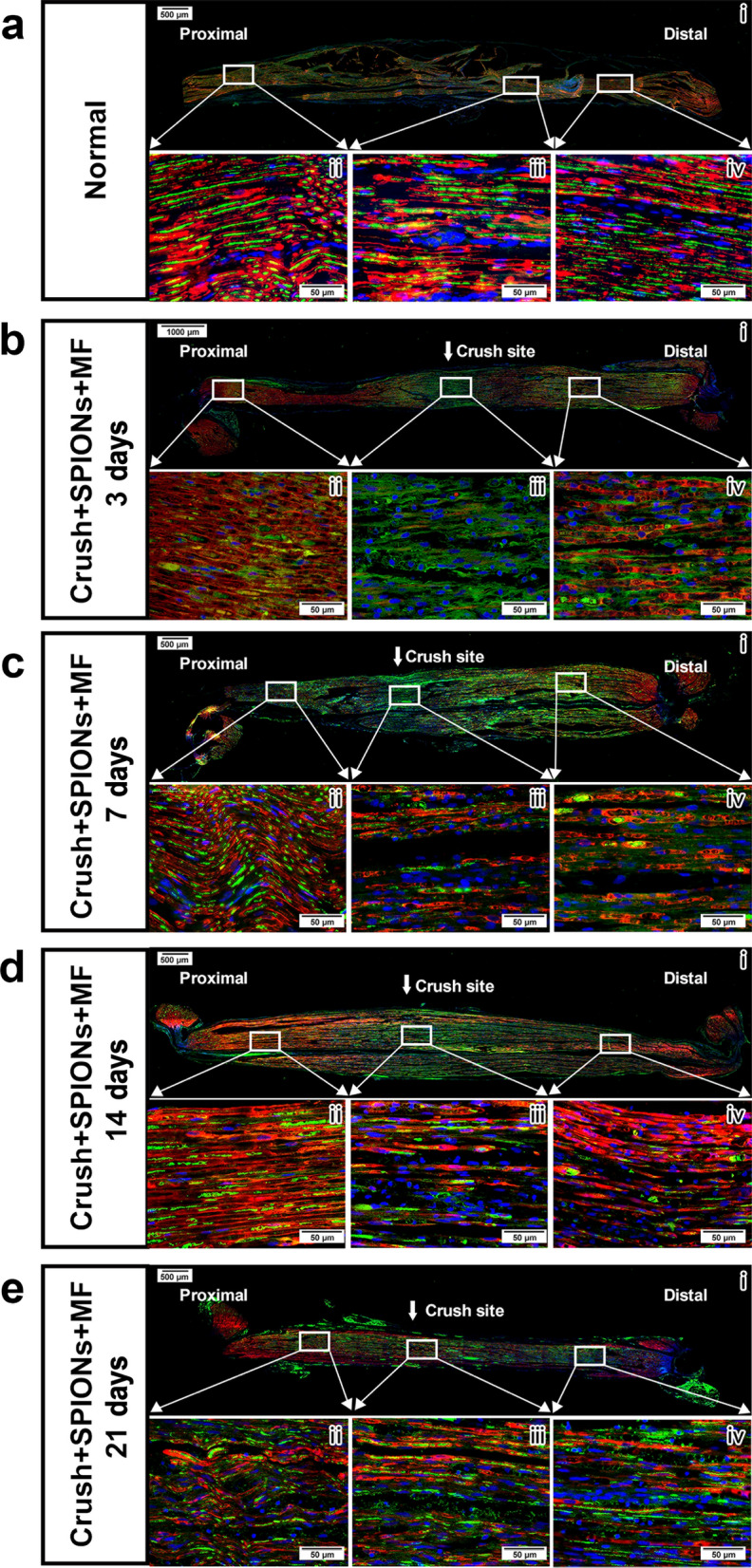
Regeneration and repair of the sciatic nerve after crush injury in the magnetic actuation group**.** The regeneration of axons (green) and the interaction of axons with Schwann cells (red) after crush injury of the sciatic nerve were observed by immunofluorescence staining using neurofilament heavy chain antibody and S100β antibody. **a** Microstructure of normal sciatic nerve. Schwann cells form myelin sheaths (red) around axons (green). **b** In the Crush + SPION + MF group, the microstructure of the sciatic nerve 3 days after crush injury showed obvious Wallerian degeneration at the crush site (**iii**) and distal end (**iv**). **c** In the Crush + SPION + MF group, the microstructure of the sciatic nerve 7 days after crush injury showed the growth of new regenerated axons at the crush site (**iii**), and the regenerated axons were not wrapped by the myelin sheath (**iv**). **d** In the Crush + SPION + MF group, the microstructure of the sciatic nerve 14 days after crush injury showed numerous regenerated axons at the crush site (**iii**), and significant remyelination was observed. Meanwhile, bands of Bungner (red) formed from Schwann cells arranged in rows were seen at the distal stump of the nerve (**iv**). **e** In the Crush + SPION + MF group, the microstructure of the sciatic nerve 21 days after crush injury showed robust axon regeneration at both the crush site (**iii**) and the distal end of the nerve (**iv**), and the regenerated axons obtained good remyelination. Proximal = proximal stump of sciatic nerve to the crush site. Distal = stump of sciatic nerve distal to the crush site

Figure 7a shows a long longitudinal section of the normal sciatic nerve, in which the green fluorescently labeled axons are surrounded by red fluorescently labeled myelin sheaths in myelinated nerve fibers. Figure 7b shows the histological appearance of the sciatic nerve 3 days after crush injury. DAPI staining showed cell proliferation at the crush site (Fig. 7b(iii)), fluorescent myelin and fluorescent neurofilament antibody staining showed myelin breakdown, cytoskeletal component degradation and axon rupture at the distal site of injury (Fig. 7b(iv)). In agreement with previous semithin sections and TEM studies (Fig. 6c), axons rapidly regenerated 7 days after nerve crush injury in the magnetic actuation group (Fig. 7c). We clearly observed new regenerated axons guided by proximal Schwann cells through the crush site (Fig. 7c(iii)). At this timepoint, the regenerated nerve fibers were not wrapped in myelin sheaths. At the distal end of the sciatic nerve crush point, typical Wallerian degeneration was observed, with severe internal structure disorganization characterized by the disintegration of nerve axons and segmentation of myelin debris into discrete ovoids (Fig. 7c(iv)).

At 14 days after sciatic nerve crush injury, significant nerve regeneration was observed in the magnetic actuation group (Fig. 7d). The new regenerated axons grew to a considerable distance from the proximal initial crush site and extended into the distal end of the nerve. These regenerated axons were covered by Schwann cells, which bound the axons together crossing the crush point (Fig. 7d(iii)). Bungner band formation was observed at the distal stump of the nerve (Fig. 7d(iv)), which were cell columns made from overlapping elongated, bipolar repair Schwann cells lying inside the basal lamina tubes that previously enclosed myelin or Remak cells and their associated axons before injury. Repairing Schwann cells organized into bands of Bungner is essential for axonal guidance during regeneration.

Twenty-one days after injury, robust axonal regeneration was observed in the magnetic actuation group, and the regenerated axons were evenly distributed from the proximal to the distal end of sciatic nerves and obtained good remyelination (Fig. 7e). By analyzing the processes of axon outgrowth from proximal to distal nerve stumps in the magnetic actuation group, we demonstrated that magnetic actuation mediated by SPIONs effectively promoted regeneration of the sciatic nerve after crush injury. Axons regenerate along the distal nerve with the help of magnetically induced repair Schwann cells and retained basal lamina tubules, which enhances axon elongation and facilitates adequate target reinnervation.

#### SFI assessment

After evaluating the role of magnetic actuation in sciatic nerve histomorphological regeneration, we further evaluated the functional consequences of SPION-mediated magnetic actuation on nerve regeneration. Before nerve injury, the mean SFI was close to 0. On days 3, 7, 14, and 21 after sciatic nerve injury, walking trajectory measurements were taken in each rat, and SFI was calculated according to Eq. (4).

**Fig. 8:**
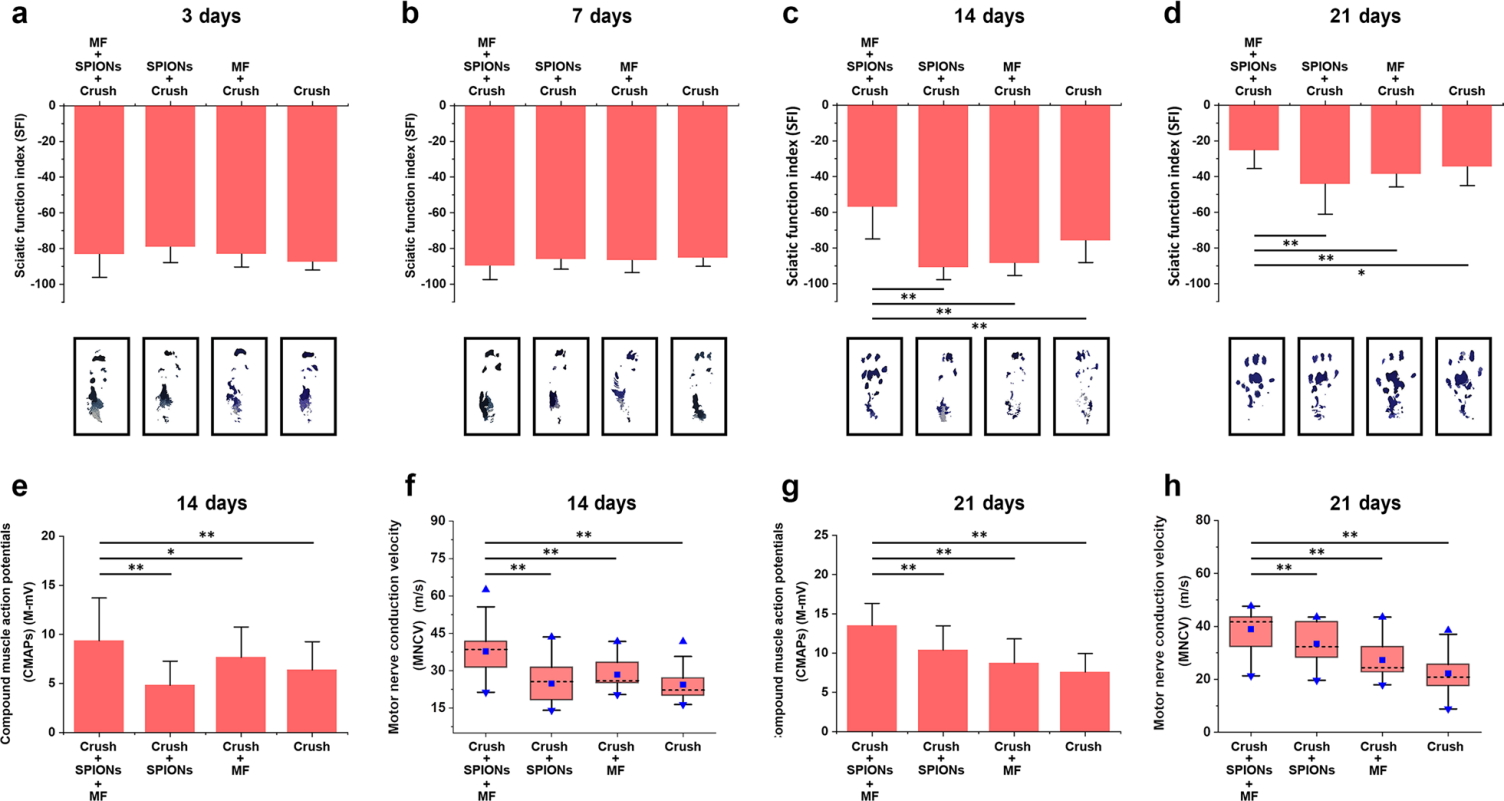
SPION-mediated magnetic actuation promotes functional and electrophysiological recovery of the sciatic nerve. The SFI was used to assess the functional recovery of nerves at 3 (**a**), 7 (**b**), 14 (**c**), and 21 days (**d**) after crush injury. The CMAPs (**e**, **g**) and MNCV (**f**, **h**) were measured using needle electromyography to assess the electrophysiological recovery of the sciatic nerve at 14 and 21 days after crush injury. The boxes in panels a-d show representative footprints. Each experiment was carried out six times. The values are represented as the mean ± SD in all panels except f and h. In panel f and h, the box plot shows the median (whiskers), interquartile ranges (boxes), and 5^th^–95^th^ percentiles (line); the square represents the mean value, and the triangles represent maximum and minimum. *P < 0.05, **P < 0.01

At 3 and 7 days after nerve crush injury, there was significant hindlimb impairment due to complete loss of sciatic nerve function, and the mean SFI decreased to near -100 across all trials (Fig. 8a, b, Additional file 3: Movie S2 and Additional file 4: Movie S3). There was no significant difference among the magnetic actuation group, SPIONs control group, MF control group and surgical control group (*p* > 0.05). We found that SFI values in the magnetic actuation group (− 57.00 ± 17.9) increased rapidly on day 14 after injury and were significantly higher than those in the SPIONs control group (− 90.90 ± 6.8), MF control group (− 88.50 ± 6.9), and surgical control group (-75.90 ± 12.2) (Fig. 8c and Additional file 5: Movie S4). These results indicated that the magnetic actuation group experienced a rapid process of nerve regeneration and functional recovery at 14 days after sciatic nerve crush injury. According to Fig. 8d and Additional file 6: Movie S5, on the 21st day after injury, SFI in all four experimental groups increased significantly, but statistical analysis showed that SFI values in the magnetic actuation group (− 25.30 ± 10.20) were significantly higher than those in the other three control groups (− 44.20 ± 16.80 in Crush + SPIONs, − 38.60 ± 7.20 in Crush + MF, and − 34.5 ± 10.50 in Crush, *P* < 0.05), suggesting better neurological function recovery. The results of the sciatic nerve function assessment were consistent with the histomorphological analysis described previously, which suggested that SPION-mediated magnetic actuation promoted functional recovery of the injured sciatic nerve, while the SFI of rats was not affected by SPIONs or MF alone.

#### Electrophysiological assessment of the sciatic nerve

In animal experiments, nerve regeneration can be evaluated on the basis of electrophysiological detection in addition to morphological and functional assessments. In the first week after nerve crush injury, no effective CMAPs were elicited in any of the experimental groups. On the 14th day after nerve crush injury, CMAPs and MNCV in the magnetic actuation group (9.37 ± 4.34 mV and 38.50 (31.30–42.15) m/s) were significantly higher than those in the SPIONs control group (4.83 ± 2.45 mV and 25.60 (18.20–31.30) m/s, *P* < 0.01), the MF control group (7.76 ± 3.08 mV and 25.95 (24.70–33.90) m/s, *P* < 0.05) and the surgical control group (6.38 ± 2.88 mV and 22.20 (19.80–28.65) m/s, *P* < 0.01) (Fig. 8e, f). This suggested that the magnetically stimulated rats experienced a rapid recovery of the electrophysiological function of the sciatic nerve at 2 weeks after injury. The same trend continued up to 3 weeks after crush injury. CMAPs and MNCV were further recovered in all experimental groups, but the magnetic actuation group showed better recovery than the other three control groups (Fig. 8g, h).

### SPION-mediated magnetic actuation induces and maintains repair-supporting phenotypes in Schwann cells during regeneration after sciatic nerve crush injury in vivo

In previous studies, we demonstrated that magnetic actuation mediated by SPIONs promoted regeneration and functional recovery of the sciatic nerve after crush injury. To explore the underlying mechanism behind this impressive phenomenon, in this part of the experiment, we examined and analyzed the characteristic phenotypes of the repair Schwann cells in the injured sciatic nerves of five experimental groups to prove the role of SPION-mediated magnetic actuation in the generation and maintenance of the repair Schwann cell phenotypes after nerve injury.

#### In vivo*, SPION-mediated magnetic actuation upregulates the expression of neurotrophic factors in Schwann cells*

**Fig. 9:**
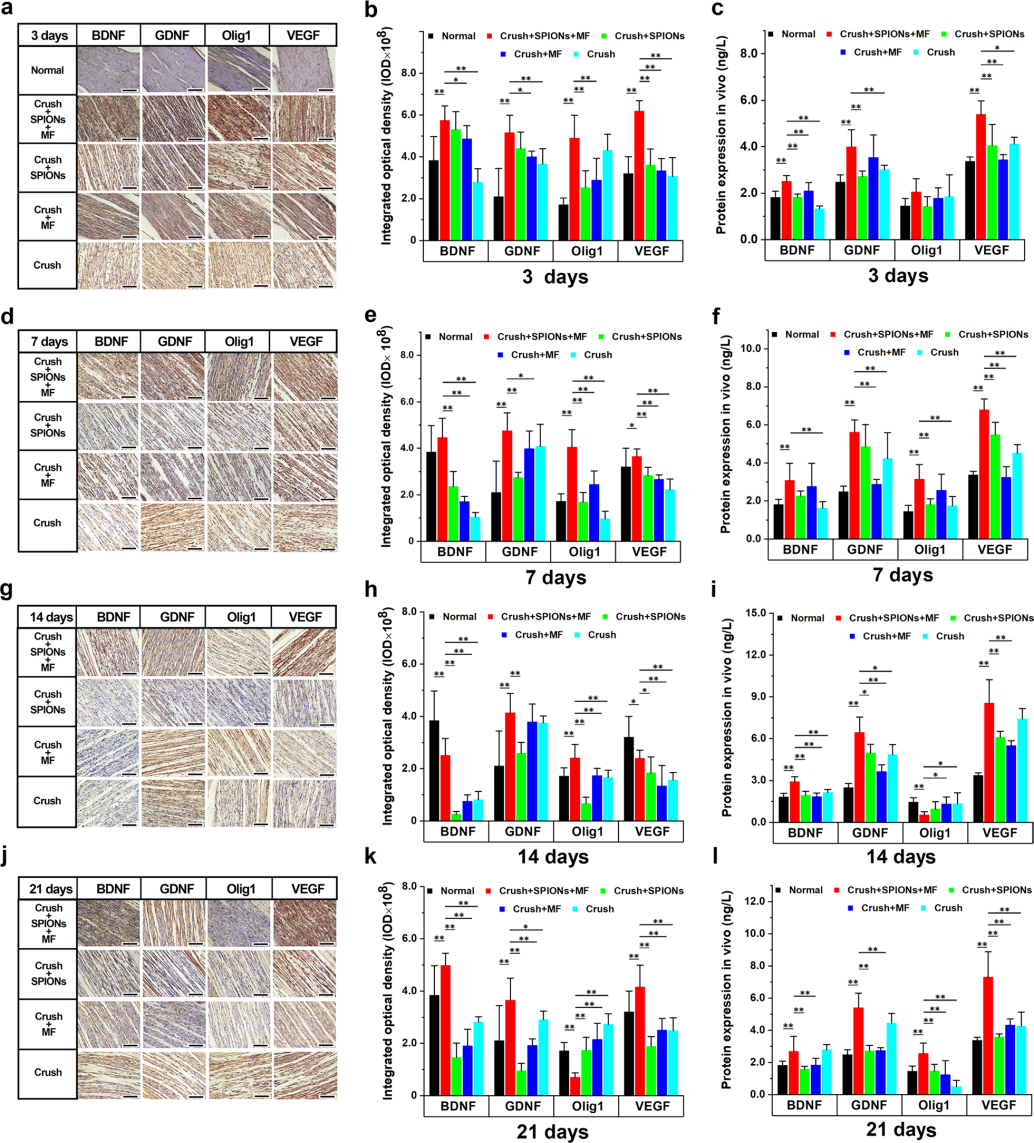
SPION-mediated magnetic actuation upregulates the expression of neurotrophic factors associated with repair phenotypes in Schwann cells. The protein expression of repair phenotype-related neurotrophic factors BDNF, GDNF, Olig1 and VEGF in different experimental groups was detected by immunohistochemical staining at 3 **(a)**, 7 **(d)**, 14 **(g)** and 21 days **(j)** after crush injury, and the protein expression levels were quantitatively analyzed (**b**, **e**, **h**, **k**). **c**, **f**, **i**, **l** The protein expression levels of such neurotrophic factors at the above time points were detected by ELISA, and the results were consistent with the immunohistochemical analysis. Each experiment was carried out in triplicate. The values are represented as the mean ± SD. Scale bar = 50 µm in panels **a**, **d**, **g**, **j**. *P < 0.05, **P < 0.01

Immunohistochemical staining and ELISA were used to detect the expression of neurotrophic factors, including BDNF, GDNF, Olig1 and VEGF, in different experimental groups at different time points after sciatic nerve crush injury. Figure 9a, d, g, and j showed the immunohistochemical images of BDNF, GDNF, Olig1 and VEGF proteins in the Crush + SPIONs + MF, Crush + SPIONs, Crush + MF, and Crush groups at 3, 7, 14, and 21 days after sciatic nerve crush injury, respectively. As shown in Fig. 9b, e, h and k, quantitative immunohistochemical analysis of the above images showed that BDNF protein expression continued to be upregulated within 3 weeks after nerve injury in the magnetic actuation group, significantly higher than that in the normal, SPIONs, MF, and surgical control groups. GDNF protein showed the same trend as BDNF, and the expression in the magnetic actuation group was significantly upregulated compared with the other four control groups at the above four time points. For Olig1 protein, similar changes were observed. Under induction of magnetic stimulation, the expression of Olig1 was continuously upregulated at 3, 7 and 14 days after crush injury and decreased at 21 days. During the 21-day observation period, VEGF expression was significantly higher in the magnetic actuation group than in the other four controls.

To further validate the immunohistochemical results, we used ELISA to detect the protein expression levels of BDNF, GDNF, Olig1 and VEGF in sciatic nerve samples from five experimental groups. As shown in Fig. 9c, f, i and l, the ELISA results were highly consistent with the quantitative immunohistochemical results. These results suggested that magnetic actuation mediated by SPIONs could induce sustained high expression of neurotrophins in vivo.

#### In vivo*, SPION-mediated magnetic actuation activates Schwann cell autophagy*

In the first stage of posttraumatic nerve regeneration, known as Wallerian degeneration, autophagy in repair Schwann cells is strongly activated, which is considered to be a key process in the transformation of Schwann cells into repair cells. Therefore, WB was used to detect the expression levels of the autophagy-related proteins Beclin1, LC3B, and p62 in sciatic nerve tissues from the five experimental groups.

**Fig. 10:**
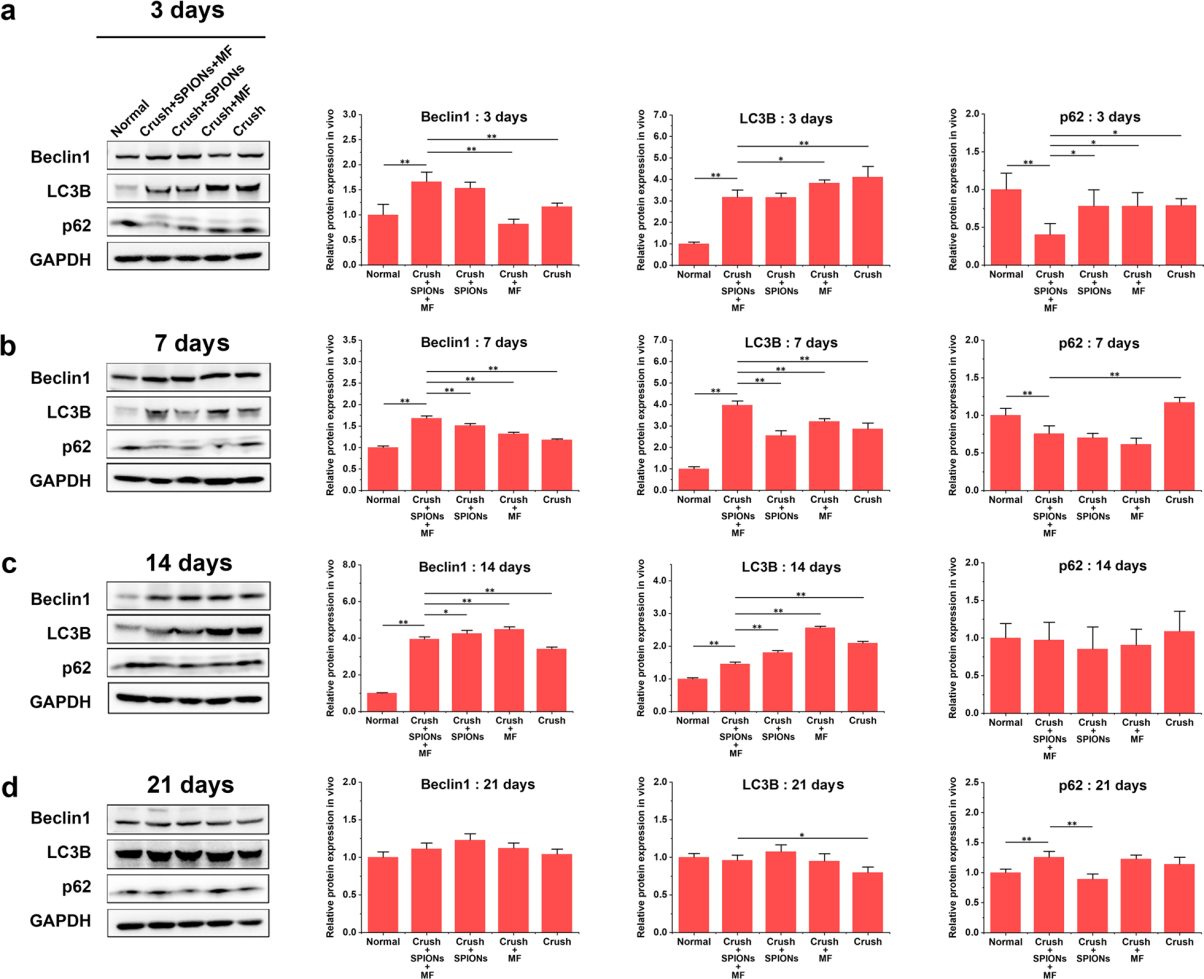
SPION-mediated magnetic actuation activates autophagy in Schwann cells. The relative protein expression of autophagy-related biomarkers (Beclin1, LC3B, and p62) in different experimental groups was detected by WB at 3 (**a**), 7 (**b**), 14 (**c**), and 21 days (**d**) after sciatic nerve crush injury to evaluate the effect of different treatments on the autophagy activity of Schwann cells. Each experiment was carried out in triplicate. The values are represented as the mean ± SD. *P < 0.05, **P < 0.01

We found that Beclin1 and LC3B protein expression was significantly increased in the magnetic actuation group at the distal end of the injured nerve in the early stage of sciatic nerve crush injury (3 days after crush injury) (Fig. 10a). Beclin1 and LC3B levels in the magnetic actuation group continued to be higher than those in the normal control, SPIONs control, MF control and surgical control groups until 7 days after crush injury (Fig. 10b). Thereafter, Beclin1 and LC3B protein expression in the magnetic actuation group was gradually downregulated and was lower than those in the SPIONs control, MF control and surgical control groups on day 14 after injury but still significantly higher than that in the normal control group (Fig. 10c). On the 21st day after injury, there was no significant difference in Beclin1 and LC3B expression between the five experimental groups (Fig. 10d). As an important substrate in autophagy, the p62 protein content in tissues is inversely proportional to autophagy activity. Within 1 week after nerve crush injury, the level of p62 decreased significantly due to autophagy activation and was significantly lower in the magnetic actuation group than in the other four control groups (Fig. 10a, b). After 2 weeks of injury, there was no significant difference in p62 levels between the five groups (Fig. 10c, d).

These results indicated that the autophagy activity of repair Schwann cells followed a temporal sequence after nerve injury. In the early stage after nerve injury, during Wallerian degeneration, SPION-mediated magnetic actuation activates autophagy in Schwann cells, which plays a major role in myelin clearance and subsequent axon regeneration. In contrast, autophagy activity was significantly reduced during the later stage of nerve injury (2 weeks after injury) to prevent excessive autophagy from damaging the remyelination of regenerated axons.

#### In vivo*, SPION-mediated magnetic actuation upregulates the expression of adhesion molecules in Schwann cells*

**Fig. 11:**
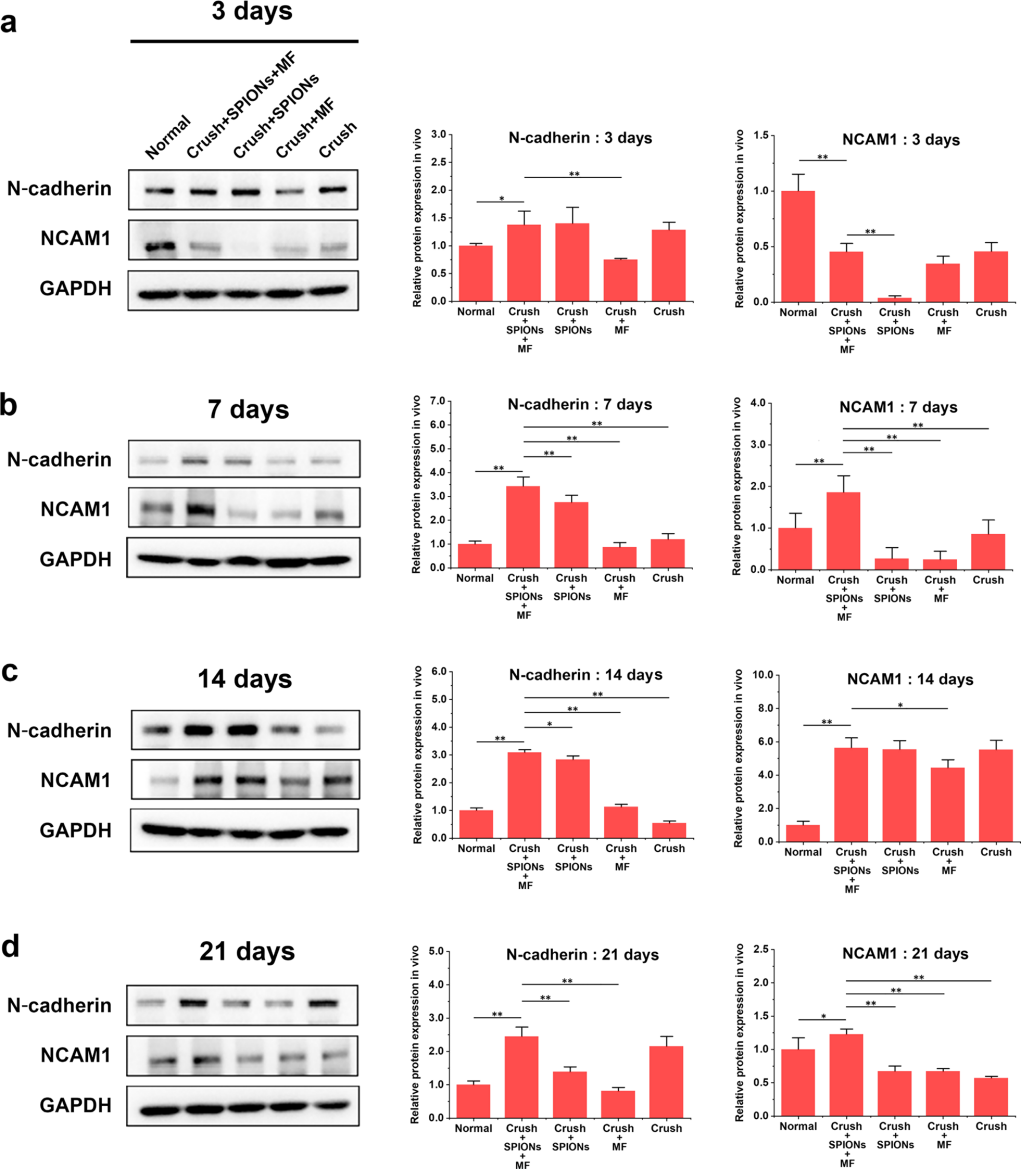
SPION-mediated magnetic actuation upregulates the expression of adhesion molecules associated with repair phenotypes in Schwann cells. The relative protein expression of N-cadherin and NCAM in different experimental groups was detected by WB at 3 (**a**), 7 (**b**), 14 (**c**), and 21 days (**d**) after crush injury of the sciatic nerve to evaluate the effect of different treatments on the expression of repair phenotype-related adhesion molecules in Schwann cells. Each experiment was carried out in triplicate. The values are represented as the mean ± SD. *P < 0.05, **P < 0.01

During regeneration after nerve injury, adhesion molecules associated with axon growth and elongation, such as N-cadherin and NCAM, are upregulated. These proteins are involved in axon–extracellular matrix and cell–cell interactions. Interactions between regenerating axons and Schwann cells through adhesion molecules play an important role in guiding axons to their target organs. We detected the expression of N-cadherin and NCAM proteins during nerve regeneration in different experimental groups through WB quantitative analysis. WB results showed that N-cadherin and NCAM expression was not significantly upregulated in any group at the early stage of nerve injury (within 3 days after injury) (Fig. 11a). However, from 7 days after injury, with nerve regeneration, the expression of the abovementioned proteins increased rapidly in the magnetic actuation group and was significantly higher than that in the normal control group, SPIONs control group, MF control group, and surgical control group (Fig. 11b). This difference persisted until the experiment was terminated at 21 days after injury (Fig. 11c, d). These results demonstrated that SPION-mediated magnetic actuation upregulated the expression of regeneration-related adhesion molecules after nerve injury in vivo.

#### In vivo*, SPION-mediated magnetic actuation promotes remyelination of regenerated nerve fibers*

**Fig. 12:**
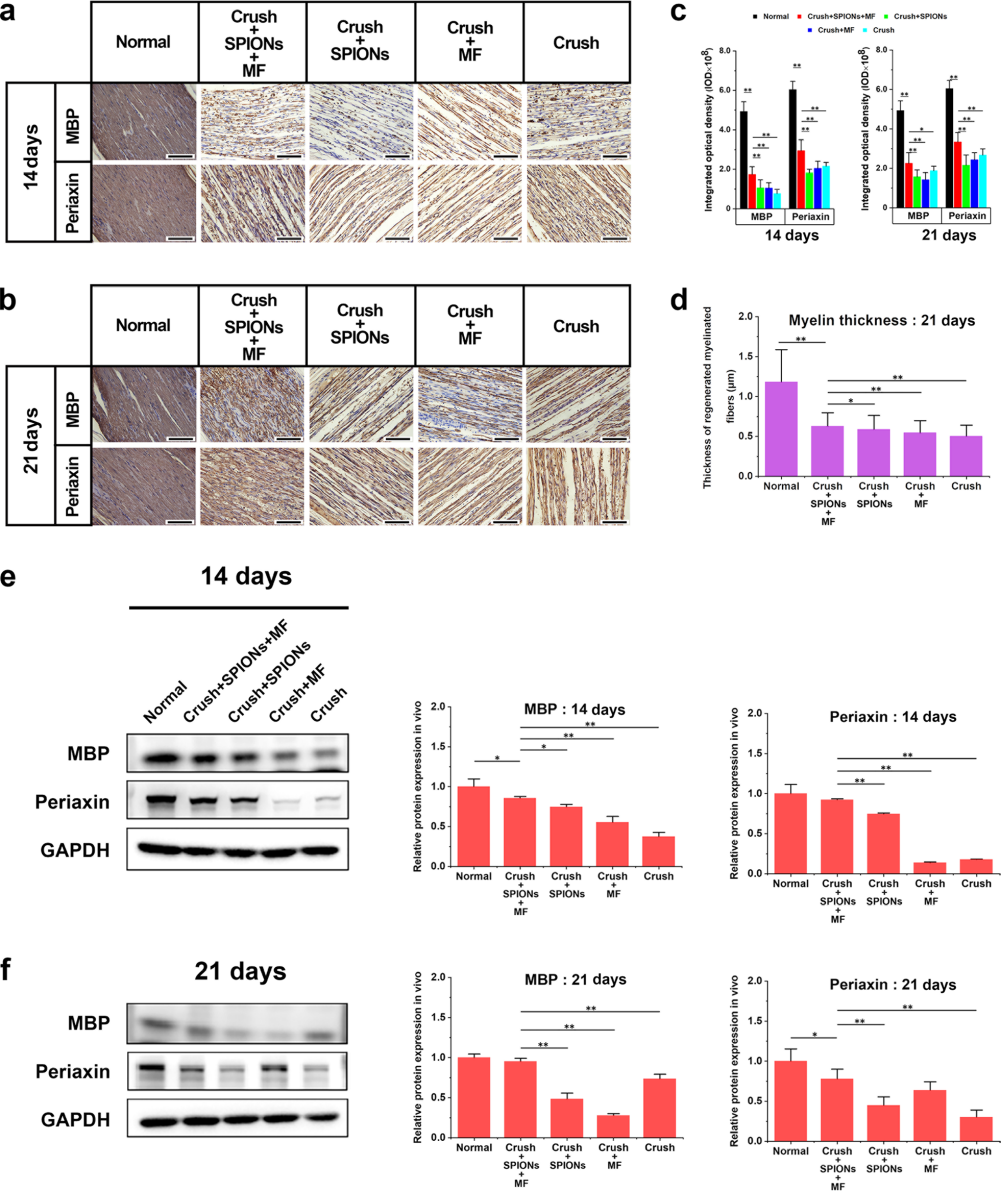
SPION-mediated magnetic actuation promotes remyelination of regenerated nerve fibers. The expression of myelin-associated structural proteins (periaxin and MBP) was detected by immunohistochemistry in different experimental groups at 14 (**a**) and 21 (**b**) days after sciatic nerve crush injury. **c** The protein expression levels in immunohistochemical images were quantitatively analyzed to evaluate the effect of different treatments on nerve remyelination. **d** The myelin thickness of regenerated myelinated nerve fibers was measured 3 weeks after nerve crush injury to evaluate the effect of different treatment factors on nerve remyelination from a morphological perspective. **e**, **f** The relative protein expression of periaxin and MBP in different experimental groups was measured by WB at the above time points to validate the immunohistochemical results. Each experiment was carried out in triplicate. The values are represented as the mean ± SD. Scale bar = 50 µm in panels a and b. *P < 0.05, **P < 0.01

At the late stage of nerve regeneration, repair Schwann cells redifferentiate into myelin Schwann cells or Remak Schwann cells and remyelinate the regenerated axons. Myelinating Schwann cells produce myelin sheaths around regenerated nerve fibers and express characteristic myelin markers. We used immunohistochemistry and WB methods to detect myelin-related proteins, including MBP and periaxin, in different experimental groups early (14 days after injury in Fig. 12a) and middle stages (21 days after injury in Fig. 12b) of nerve regeneration. Immunohistochemical quantitative results in Fig. 12c showed that MBP and periaxin protein expression levels were significantly increased in the magnetic actuation group at 14 and 21 days after sciatic nerve crush injury. To further verify these results, we measured the amounts of these two myelin-related structural proteins in the sciatic nerve using WB. The results were consistent with immunohistochemistry, and the expression of MBP and periaxin proteins was significantly higher in the magnetic actuation group than in the SPIONs, MF, and surgical controls (Fig. 12e, f). Consistent with this, myelin thickness of myelinated nerve fibers was measured by TEM at 3 weeks after nerve injury, and the results showed that the myelin thickness of the magnetic actuation group was significantly higher than that of the other three controls, although still smaller than normal control group (Fig. 12d).

These results suggested that magnetic actuation mediated by SPIONs can promote the remyelination of regenerated axons during the period of nerve regeneration.

### SPION-mediated magnetic actuation activates transcriptional regulation and signaling pathways associated with Schwann cell repair phenotypes in vivo

We have proven that SPION-mediated magnetic actuation induces and maintains Schwann cell repair-supporting phenotypes that promote peripheral nerve regeneration by previous results. However, a key question is how SPION-mediated mechanical signals are transduced into intracellular biochemical signals that regulate phenotypic transformation. To answer this question, we need to introduce the concept of mechanotransduction. Mechanotransduction is the initiation of cellular activity via intracellular biochemical signal transduction through mechanical forces applied to cells, including compression, tension, hydrodynamic force and magnetism [26, 55, 56]. Therefore, there is great potential to use this phenomenon to manipulate the gene expression pattern of denervated Schwann cells after nerve injury through exogenous manipulation to obtain and control the functional phenotypes in regenerative medicine [26]. Magnetic actuation is a promising method for performing this type of exogenous manipulation on therapeutic cells. Our previous results have demonstrated that magnetic actuation can influence the repair behaviors of Schwann cells.

To further explore the intracellular mechanotransduction mechanisms of SPION-mediated magnetic actuation regulating the repair phenotypes of Schwann cells, we investigated the transcription factors and their upstream signaling pathways associated with repair phenotypes under magnetic actuation. The most important transcriptional mechanisms involved in the generation and maintenance of repair Schwann cells are c-Jun and STAT3.

The transcription factor c-Jun is essential for the normal activation of the repair program and acts as a key regulator of the Schwann cell injury response [5, 15]. The c-Jun expression is rapidly induced at high levels in the Schwann cells of injured nerves and promotes expression of the repair program, which includes an increase in neurotrophic support for neurons, acceleration of myelin breakdown by autophagy, promotion of Schwann cell elongation, and formation of the bands of Bungner [12, 15, 57–61]. Recently, the transcription factor STAT3 has also been shown to be involved in the long-term maintenance of repair Schwann cells [62]. During chronic denervation, injury triggers continuous activation of STAT3 in Schwann cells. In Schwann cells with selective inactivation of STAT3, there were fewer Schwann cells, reduced expression levels of repair cell markers and neurotrophic factors, and structurally distorted bands of Bungner. In contrast, Schwann cells developed normally when STAT3 expression was upregulated. This suggests that STAT3 plays a special role in the Schwann cell injury response by supporting the survival of chronically denervated Schwann cells and maintains their long-term molecular and morphological differentiation.

**Fig. 13:**
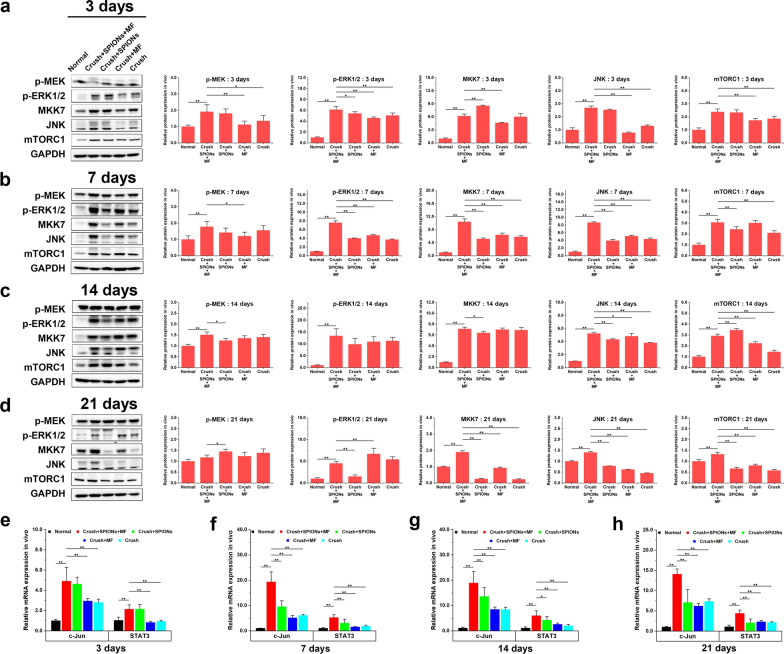
SPION-mediated magnetic actuation upregulates transcription factor expression and activates signaling pathways associated with Schwann cell repair phenotypes. The expression levels of biomarkers of the Raf-MEK-ERK pathway (p-MEK and p-ERK1/2), Rac-MKK7-JNK pathway (MKK7 and JNK) and mTORC1 pathway (mTORC1) in different experimental groups were detected by WB at 3 (**a**), 7 (**b**), 14 (**c**) and 21 days (**d**) after sciatic nerve crush injury to evaluate the effect of different treatments on repair phenotype-related signaling pathways in Schwann cells. The relative mRNA expression levels of transcription factors (c-Jun and STAT3) in different experimental groups were measured by qRT-PCR at 3 (**e**), 7 (**f**), 14 (**g**) and 21 days (**h**) after sciatic nerve crush injury to evaluate the effect of different treatments on repair phenotype-related transcription factors in Schwann cells. The relative mRNA expression was calculated by using the 2-ΔΔCT method. Each experiment was carried out in triplicate. The values are represented as the mean ± SD. *P < 0.05, **P < 0.01

We used qRT-PCR to detect the expression of these transcription factors in different experimental groups within three weeks after nerve injury. As shown in Fig. 13e–h, the expression of the transcription factors c-Jun and STAT3 was continuously upregulated in the magnetic actuation group during a 3-week observation period after sciatic nerve crush injury. Compared with normal controls, sciatic nerve crush injury induced upregulation of c-Jun and STAT3 in magnetic actuation, SPIONs, MF application alone, and surgical controls. However, the relative mRNA expression levels of these transcription factors in the magnetic actuation group were significantly higher than those in the other four control groups (*P* < 0.05), and this high expression pattern remained stable for up to 3 weeks after injury (Fig. 13e–h).

At the same time, several upstream activation pathways have been identified that may regulate the repair phenotypes of Schwann cells after nerve injury, including the Raf-MEK-ERK, Rac1-MKK7-JNK and mTORC1 pathways [53]. ERK1/2 phosphorylation is rapidly induced in Schwann cells after injury and participates in macrophage recruitment by Schwann cells, while activation of the Rac1-MKK7-JNK and mTORC1 pathways promotes c-Jun and regeneration-related gene translation.

We used WB to detect the activation status of the abovementioned signaling pathways that are closely related to Schwann cell repair phenotypes. The expression and phosphorylation enrichment levels of the main biomarker proteins were quantitatively analyzed. As shown in Fig. 13a, b, phosphorylated MEK (p-MEK) and ERK1/2 (p-ERK1/2) were significantly upregulated in the magnetic actuation group within 7 days after nerve injury and were significantly higher than those in the other controls. This suggested that SPIONs mediated magnetic actuation induced MEK and ERK1/2 phosphorylation and activated the Raf-MEK-ERK signaling pathway in Schwann cells in the early stage of nerve injury. The Raf-MEK-ERK pathway is speculated to control the immune function of Schwann cells, which is closely related to the autophagy activity of Schwann cells. This result was consistent with the previous conclusion that SPION-mediated magnetic actuation activated autophagy in Schwann cells during the early stage of Wallerian degeneration after nerve injury.

According to Fig. 13a–d, the protein expression levels of MKK7, JNK and mTORC1 were significantly upregulated in the magnetic actuation group during the observation period of three weeks after sciatic nerve crush injury and were significantly higher than those in the other controls, which suggested that SPION-mediated magnetic actuation induced sustained activation of the Rac-MKK7-JNK and mTORC1 pathways. Activation of these pathways promotes the translation of c-Jun and the expression of a series of regeneration-related genes. These results were consistent with previous findings that SPION-mediated magnetic actuation induced sustained high expression of neurotrophic factors and regeneration-related adhesion molecules after nerve injury.

## Conclusion

In this study, we applied SPIONs to sciatic nerves and established an effective MF stimulation system for magnetic actuation of Schwann cells. Our research points toward a promising phenomenon: with the use of SPIONs as magnetic stimulation actuators, extracellular mechanical force signals can be converted to intracellular biochemical signals, and transcriptional regulation of repair-supporting phenotypes can subsequently be initiated through the activation of relevant signaling pathways, which ultimately promotes peripheral nerve regeneration by inducing and maintaining repair phenotypes in Schwann cells. This study demonstrates that SPION-mediated magnetic actuation can be an effective therapeutic tool for manipulating the phenotypes and behaviors of therapeutic cells in the field of regenerative medicine. We hope that this study will provide a new therapeutic strategy for the regeneration and repair of peripheral nerve injury.

## Future perspective

Applications of magnetic actuation technologies have great potential in furthering regenerative medicine. The following three issues need to be addressed in future research. (1) Functionalization of magnetic nanoparticles: in order to have a wider and deeper application in the biomedical field, it is inevitable to properly functionalize nanoparticles. Magnetic nanoparticles can be physically or chemically modified by different biomolecules, such as enzymes, drugs, antibodies, proteins, nucleic acids, etc., which allows functionalized nanoparticles to interact specifically with target cells or subcellular structures. (2) Temporal and spatial specificity of magnetic actuation: the response of cells to external mechanical stimuli is a complex process, which depends not only on the magnitude of force, but also on the rate of force loading and the frequency of force applied. At the same time, the timescales of the externally applied forces need to match the intrinsic timescales of the target intracellular signaling processes, for the intended mechanical control of biological phenomena to occur. In fact, it has been speculated that the cellular response to physical stimuli may be as complex as its biochemical and genetic signaling pathways. Recent studies have demonstrated that mechanical stresses may convey large amounts of information through precise time-dependent and spatially-dependent modulation. A major direction of future research is to further study the temporal and spatial specificity of magnetic actuation. (3) Mechanism of mechanotransduction: the effect of mechanical forces in the development and regeneration of the nervous system has been increasingly recognized and addressed. At present, a key question is how mechanical stimuli from the outside are transduced into biochemical signals for nerve regeneration, which involves the concept of "Mechanotransduction". Mechanotransduction, the conversion of mechanical stimuli into biochemical signaling, contributes to numerous developmental and physiological processes. In order to further clarify this unknown mechanism of mechanotransduction, it is essential to analyze gene and protein expression profiles using genomics and proteomics techniques by integrating the best aspects of biophysics, genetics, and cell biology.

## Supplementary Information

**Additional file 1:** Supporting Information.

**Additional file 2:** **Movie S1.** SPION-mediated magnetic actuation induces RSC96 cells elongation and branching. RSC96 cells maintain active mitotic proliferation after endocytosis of SPIONs. The circles highlight those proliferating cells. The arrows highlight those elongated and branching cells. Video was taken over 10.5 h and time-lapsed images were collected with a per frame period of 5 min.

**Additional file 3:** **Movie S2.** On days 3 after sciatic nerve crush injury, representative walking trajectories were recorded in the magnetic actuation group (Crush + SPIONs + MF), SPIONs control group (Crush + SPIONs), MF control group (Crush + MF) and surgical control group (Crush). E = experimental side, N = normal side.

**Additional file 4:** **Movie S3.** On days 7 after sciatic nerve crush injury, representative walking trajectories were recorded in the magnetic actuation group (Crush + SPIONs + MF), SPIONs control group (Crush + SPIONs), MF control group (Crush + MF) and surgical control group (Crush). E = experimental side, N = normal side.

**Additional file 5:** **Movie S4.** On days 14 after sciatic nerve crush injury, representative walking trajectories were recorded in the magnetic actuation group (Crush + SPIONs + MF), SPIONs control group (Crush + SPIONs), MF control group (Crush + MF) and surgical control group (Crush). E = experimental side, N = normal side.

**Additional file 6:** **Movie S5.** On days 21 after sciatic nerve crush injury, representative walking trajectories were recorded in the magnetic actuation group (Crush + SPIONs + MF), SPIONs control group (Crush + SPIONs), MF control group (Crush + MF) and surgical control group (Crush). E = experimental side, N = normal side.
