# Supplementary material for: SPIONs mediated magnetic actuation promotes nerve regeneration by inducing and maintaining repair-supportive phenotypes in Schwann cells
Source: J Nanobiotechnology. 2022 Mar 27;20:159. doi: 10.1186/s12951-022-01337-5 (PMC8966266; doi:10.1186/s12951-022-01337-5)
Supplement: Supplementary file 1 — Additional file 1: Supporting Information. [file 12951_2022_1337_MOESM1_ESM.doc]

**Supporting Information**

**SPIONs mediated magnetic actuation promotes nerve regeneration by inducing and maintaining repair-supportive phenotypes in Schwann cells**

Ting Liu^1^, Yang Wang^2^*, Laijin Lu^2^, Yi Liu^3^*

1. Department of Geriatrics, the First Hospital of Jilin University, Changchun 130021, P. R. China.

2. Department of Hand Surgery, the First Hospital of Jilin University, Changchun 130021, P. R. China.

3. State Key Laboratory of Supramolecular Structure and Materials, College of Chemistry, Jilin University, Changchun 130012, P. R. China.

* Corresponding Authors: Yang Wang & Yi Liu.

Address correspondence to [wangy19851022@jlu.edu.cn](mailto:wangy19851022@jlu.edu.cn); yiliuchem@jlu.edu.cn

**Table of the Electronic Supporting Information**

**(1) Synthesis and characterization of fluorescent-magnetic SPIONs**

**(2)** **Quantitative real-time PCR (qRT-PCR) analysis**

**(3) Table S1.** Sequences of the primers used in this study.

**(4) ELISA analysis**

**(5) Quantification of internal SPIONs per cell**

**(6) Figure S1.** Morphological regeneration of the sciatic nerve in the SPIONs control group

**(7) Figure S2.** Morphological regeneration of the sciatic nerve in the MF control group

**(8) Figure S3.** Morphological regeneration of the sciatic nerve in the surgical control group

**(9) Movie S1.** SPION-mediated magnetic actuation induces RSC96 cells elongation and branching.

**(10) Movie S2.** On days 3 after sciatic nerve crush injury, representative walking trajectory in the the magnetic actuation group (Crush+SPIONs+MF), SPIONs control group (Crush+SPIONs), MF control group (Crush+MF) and surgical control group (Crush).

**(11) Movie S3.** On days 7 after sciatic nerve crush injury, representative walking trajectory in the the magnetic actuation group (Crush+SPIONs+MF), SPIONs control group (Crush+SPIONs), MF control group (Crush+MF) and surgical control group (Crush).

**(12) Movie S4.** On days 14 after sciatic nerve crush injury, representative walking trajectory in the the magnetic actuation group (Crush+SPIONs+MF), SPIONs control group (Crush+SPIONs), MF control group (Crush+MF) and surgical control group (Crush).

**(13) Movie S5.** On days 21 after sciatic nerve crush injury, representative walking trajectory in the the magnetic actuation group (Crush+SPIONs+MF), SPIONs control group (Crush+SPIONs), MF control group (Crush+MF) and surgical control group (Crush).

**(14) Abbreviations**

**(15) References**

**(1)** **Synthesis and characterization of** **fluorescent-magnetic SPIONs**

The fluorescent-magnetic bifunctional SPIONs used in our study (Fe_3_O_4_·Rhodamine 6G@polydopamine superparticles) were prepared following our previously established protocol. In brief, oleic acid (OA)-capped Fe_3_O_4_ nanoparticles (NPs) were synthesized following the thermal decomposition method [1]. The average diameter of the as-prepared Fe_3_O_4_ NPs was 5.8 nm. Subsequently, OA-capped Fe_3_O_4_ NPs dispersed in toluene and water containing sodium dodecyl sulfate (SDS) and Rhodamine 6G were mixed to form an oil-in-water (O/W) microemulsion. Fe_3_O_4_ superparticles (SPs) with an average diameter of 50 nm were produced after the evaporation of toluene [2, 3]. Finally, polydopamine (PDA) was coated on the surface of Fe_3_O_4_·Rhodamine 6G SPs via the oxidation polymerization of dopamine monomers under alkaline conditions [4].

The average size, distribution, and morphology of SPIONs were studied by high-resolution transmission electron microscopy (HRTEM) using a Hitachi H-800 electron microscope at an acceleration voltage of 200 kV with a charge-coupled device (CCD) camera. The hydrodynamic size and zeta potential of the SPIONs were measured through dynamic light scattering (DLS) by a Zetasizer Nano-ZS (Malvern Instruments). Magnetic property characterization of the samples was performed at 300 K in a superconducting quantum interference device (SQUID) magnetometer (MPMS-XL, Quantum Design, Inc., San Diego, CA). Fluorescence spectroscopy was performed with a Shimadzu RF-5301 PC spectrophotometer.

**(2) Quantitative real-time PCR (qRT-PCR) analysis**

Cell and nerve samples from different groups were washed three times with precooled PBS. After freezing and grinding, 1 mL of TRIzol was added to every 50 mg of tissue, and total RNA of RSC96 cells and sciatic nerve tissues was extracted. Chloroform was added for phase separation. RNA was precipitated with isopropanol and washed with 75% ethanol solution. The obtained RNA was reverse transcribed to prepare complementary DNA (cDNA) using PrimeScript RT Master Mix (Takara, Dalian, China), followed by PCR amplification. PCR was used to quantify differences in mRNA expression using the PowerUp SYBR™ Green Master Mix Kit (Waltham, MA, USA). GAPDH was applied as an internal control. The primers used in PCR were synthesized by Invitrogen (Shanghai, China), and the sequences are listed in **Table S1.** PCR conditions were as follows: initial denaturation at 95 °C for 10 min; 95 °C for 10 s, 60 °C for 30 s, and 72 °C for 1 min, and amplification was performed for 40 cycles, followed by extension at 72 °C for 10 min. The relative mRNA expression in different groups was calculated by using the 2-ΔΔCT method. Each reaction was performed three times.

**(3) Table S1. Sequences of the primers used in this study**

| Primer | Forward primer (5'-3') | Reversed primer (5'-3') |
| --- | --- | --- |
| BDNF | TAGCGAGTGGGTCACAGCG | CGATTGGGTAGTTCGGCATT |
| GDNF | CAGAGGGAAAGGTCGCAGAG | TCGTAGCCCAAACCCAAGTC |
| Olig 1 | AGGCAGCCACCCATCTCGT | GCGGAGCTTCAGGCTTCTCA |
| VEGF | GGGCTGCTGCAATGATGAA | GTGCTGGCTTTGGTGAGGTT |
| c-Jun | CGCCAACCTCAGCAACTTCA | CGTCTGCGGCTCTTCCTTC |
| STAT3 | TTAACATTCTGGGCACGAACA | CACCACGACTGGCAAGGAG |
| GAPDH | AGACAGCCGCATCTTCTTGT | CTTGCCGTGGGTAGAGTCAT |

**(4)** **ELISA analysis**

Total proteins from different groups were extracted by RIPA lysis buffer, and the protein content was quantified by the BCA method as previously described. Protein concentrations of IL-1α, IL-1β, LIF, TNF-α, BDNF, GDNF, Olig1 and VEGF were detected using an ELISA kit (Huyu Biological Technology, Shanghai, China). Standard samples of different concentrations were added into the designated wells, with 100 μL supernatant per well. The holes were sealed with plastic membrane for 120 min of incubation at room temperature. The mixture of 50 μL sample and 50 μL analytical buffer was incubated for 120 min at room temperature. After the addition of biotinylated antibody at 100 μL per well, the wells were sealed with plastic film, and the mixture was incubated for 20 min at room temperature in the dark. Tris-buffered saline solution (100 μL) was added to each well after 5 washes. The holes were sealed with plastic film, and the resulting mixture was incubated at room temperature for 20 min in the dark. Then, 100 μL stop solution was added to each well when the color of the resulting mixture changed obviously. After slight shaking, the absorbance value at 450 nm was immediately measured. Since the concentration of target protein is directly proportional to the absorbance at 450 nm, the concentration of target protein can be calculated by measuring the absorbance value at 450 nm by ELISA.

**(5) Quantification of internal SPIONs per cell**

Spherical Fe_3_O_4_ nanoparticles (NPs) with diameter of 5.8 nm and Rhodamine 6G fluorescent molecules were selected as the building blocks to construct Fe_3_O_4_·Rhodamine 6G superparticles (SPs). Dopamine (DA) monomers can be oxidized and spontaneously polymerize to form a polydopamine (PDA) shell on the surface of Fe_3_O_4_·Rhodamine 6G SPs under alkaline conditions. Transmission electron microscopy (TEM) image show that SPIONs have an uniform size distribution, with a Fe_3_O_4_·Rhodamine 6G SPs core size of ~50 nm and a thin PDA shell of ~6 nm. According to the volume formula of the sphere (V=4/3πr^3), the volume of the SPs core is ~5.79×10^-17^ cm^3^, in which Fe_3_O_4_ NPs accounts for about 80% of the core volume, ~4.63×10^-17^ cm^3^). The density of Fe_3_O_4_ is 5.18 g/cm^3^, from which we can calculate that the mass of Fe_3_O_4_ in each SPION is ~2.40×10^-4^ pg/SPION. Based on the molar mass percentage of Fe in Fe_3_O_4_, the mass of Fe in each SPION calculates to be ~1.73×10^-4^ pg/SPION.

The RSC96 cells were seeded in 6-well plates (5 × 10^5^ cells per well) incubated 24 h. After that, the cells were treated with SPIONs (15 μg/mL) for 24 h, and washed twice with cold PBS containing deferoxamine in a concentration of 1 mM to exclude the iron that was non-specifically attached to the cell membranes rather than taken up into the cells. Then the cells were collected and counted. The amount of iron inside the cells in samples was measured by ICP-AES measurements with a PerkinElmer Optima 3300DV. Based on the number of cells in the samples we can calculate that the RSC96 cells are able to incorporate quantities of Fe up to 4.49 ± 0.27 pg/cell**.**

The number of SPIONs in each cell (*nSPIONs cell*) can be obtained by dividing the mass of iron in each cell by the mass of iron in each SPION. According to our calculation results, after incubation with the SPIONs at a concentration of 15 μg/mL, the number of SPIONs taken up by the RSC96 cells reached 2.59 ± 0.15×10^4^ SPIONs per cell.

**(6) Figure S1.** **Morphological regeneration of the sciatic nerve in** **the SPIONs control group** At 3, 7, 14, and 21 days after sciatic nerve crush injury, the semithin sections and ultrathin sections were observed by optical microscopy and transmission electron microscopy. **(a)** In the SPIONs control (Crush+SPIONs) group, the microstructure of the sciatic nerve at 3 days after crush injury were observed and showed obvious Wallerian degeneration. Myelin collapsed, and the nerve fibers were divided by myelin into early ovoid structures (P-type fibers). **(b)** In the Crush+SPIONs group, the microstructure of the sciatic nerve at 7 days after crush injury were observed and showed demyelinating nerve fibers (D-type fibers), which are filled with dense and even folded and curled myelin structures. **(c)** In the Crush+SPIONs group, the microstructure of the sciatic nerve at 14 days after crush injury were observed and showed new regenerated nerve fibers. **(d)** In the Crush+SPIONs group, the microstructure of the sciatic nerve at 21 days after crush injury were observed, and the diameter and the thickness of the myelin sheath of regenerated nerve fibers increased

**(7) Figure S2.** **Morphological regeneration of the sciatic nerve in the** **MF control group** At 3, 7, 14, and 21 days after sciatic nerve crush injury, the semithin sections and ultrathin sections were observed by optical microscopy and transmission electron microscopy. **(a)** In the MF control (Crush+MF) group, the microstructure of the sciatic nerve at 3 days after crush injury were observed and showed obvious Wallerian degeneration. Myelin collapsed, and the nerve fibers were divided by myelin into early ovoid structures (P-type fibers). **(b)** In the Crush+MF group, the microstructure of the sciatic nerve at 7 days after crush injury were observed and showed demyelinating nerve fibers (D-type fibers), which are filled with dense and even folded and curled myelin structures. **(c)** In the Crush+MF group, the microstructure of the sciatic nerve at 14 days after crush injury were observed and showed new regenerated nerve fibers. **(d)** In the Crush+MF group, the microstructure of the sciatic nerve at 21 days after crush injury were observed, and the diameter and the thickness of the myelin sheath of regenerated nerve fibers increased.

**(8) Figure S3.** **Morphological regeneration of the sciatic nerve in the** **surgical control group** At 3, 7, 14, and 21 days after sciatic nerve crush injury, the semithin sections and ultrathin sections were observed by optical microscopy and transmission electron microscopy. **(a)** In the surgical control (Crush) group, the microstructure of the sciatic nerve at 3 days after crush injury were observed and showed obvious Wallerian degeneration. Myelin collapsed, and the nerve fibers were divided by myelin into early ovoid structures (P-type fibers). **(b)** In the Crush group, the microstructure of the sciatic nerve at 7 days after crush injury were observed and showed demyelinating nerve fibers (D-type fibers), which are filled with dense and even folded and curled myelin structures. **(c)** In the Crush group, the microstructure of the sciatic nerve at 14 days after crush injury were observed and showed new regenerated nerve fibers. **(d)** In the Crush group, the microstructure of the sciatic nerve at 21 days after crush injury were observed, and the diameter and the thickness of the myelin sheath of regenerated nerve fibers increased.

**(9) Movie S1.** SPION-mediated magnetic actuation induces RSC96 cells elongation and branching. RSC96 cells maintain active mitotic proliferation after endocytosis of SPIONs. The circles highlight those proliferating cells. The arrows highlight those elongated and branching cells. Video was taken over 10.5 h and time-lapsed images were collected with a per frame period of 5 min.

**(10) Movie S2.** On days 3 after sciatic nerve crush injury, representative walking trajectories were recorded in the magnetic actuation group (Crush+SPIONs+MF), SPIONs control group (Crush+SPIONs), MF control group (Crush+MF) and surgical control group (Crush). E=experimental side, N=normal side.

**(11) Movie S3.** On days 7 after sciatic nerve crush injury, representative walking trajectories were recorded in the magnetic actuation group (Crush+SPIONs+MF), SPIONs control group (Crush+SPIONs), MF control group (Crush+MF) and surgical control group (Crush). E=experimental side, N=normal side.

**(12) Movie S4.** On days 14 after sciatic nerve crush injury, representative walking trajectories were recorded in the magnetic actuation group (Crush+SPIONs+MF), SPIONs control group (Crush+SPIONs), MF control group (Crush+MF) and surgical control group (Crush). E=experimental side, N=normal side.

**(13) Movie S5.** On days 21 after sciatic nerve crush injury, representative walking trajectories were recorded in the magnetic actuation group (Crush+SPIONs+MF), SPIONs control group (Crush+SPIONs), MF control group (Crush+MF) and surgical control group (Crush). E=experimental side, N=normal side.

**(14) Abbreviations**

BDNF: brain-derived neurotrophic factor; CCD: charge-coupled device; cDNA: complementary DNA; CLSM: confocal laser scanning microscopy; DLS: dynamic light scattering; GDNF: glial cell line-derived neurotrophic factor; HRTEM: high-resolution transmission electron microscopy; HRTEM: high resolution transmission electron microscopy; IL-1β: interleukin-1β; IL-1α: interleukin-1α; LIF: leukaemia inhibitory factor; M_s_: saturation magnetization; Olig 1: oligodendrocyte transcription factor 1; O/W: oil-in-water; PDA: polydopamine; PDA: polydopamine; qRT-PCR: quantitative real-time PCR; SDS: sodium dodecyl sulfate; SPs: superparticles; SQUID: superconducting quantum interference device; STAT3: signal transducer and activator of transcription-3; TEM: transmission electron microscopy; TNFα: tumor necrosis factor-alpha; VEGF: vascular endothelial growth factor.

**(15) References**

**[1]** Sun S, Zeng H. Size-controlled synthesis of magnetite nanoparticles. J Am Chem Soc. 2002; 124: 8204–5.

**[2]** Zhuang J, Wu H, Yang Y, Cao YC. Controlling colloidal superparticle growth through solvophobic interactions. Angew Chem Int Ed Engl. 2008; 47: 2208–12.

**[3]** Bai F, Wang D, Huo Z, Chen W, Liu L, Liang X, et al. A versatile bottom-up assembly approach to colloidal spheres from nanocrystals. Angew Chem Int Ed Engl. 2007; 46: 6650–3.

**[4]** Tang L, Yang X, Yin Q, Cai K, Wang H, Chaudhury I, et al. Investigating the optimal size of anticancer nanomedicine. Proc Natl Acad Sci USA. 2014; 111: 15344–9.
